# Consensus Guidelines for Diagnosis and Management of Pyruvate Dehydrogenase Complex Deficiency

**DOI:** 10.1002/jimd.70226

**Published:** 2026-10-02

**Authors:** Nandaki Keshavan, Julia Neugebauer, Marcello Bellusci, Enrico Bertini, Garry Brown, Niklas Darin, Suzanne DeBrosse, Lucy Drexler, Stefan Drexler, Gregory M. Enns, Rebecca D. Ganetzky, Kelly Gilbert, Austin Larson, Rebecca Legi, April N. Lehman, Camile Newby, Manuel Schiff, Rachel Skeath, Peter W. Stacpoole, Sarah Thompson, Emma Watt, Saskia B. Wortmann, Jirair K. Bedoyan, Shamima Rahman

**Affiliations:** ^1^ Metabolic Unit, Great Ormond Street Hospital NHS Foundation Trust London UK; ^2^ Mitochondrial Research Group, UCL Great Ormond Street Institute of Child Health London UK; ^3^ Department of Paediatric Gastroenterology, Nephrology and Metabolic Medicine Charité – Universitaetsmedizin Berlin Berlin Germany; ^4^ Center for Chronically Sick Children, Charité – Universitaetsmedizin Berlin Berlin Germany; ^5^ Reference Center for Inherited Metabolic Disorders MetabERN, Mitochondrial Disorders Research Group, ‘12 de Octubre’ University Hospital Madrid Spain; ^6^ Research Unit of Neuromuscular and Neurodegenerative Disease, Translational Pediatrics and Clinical Genetics Bambino Gesu Children's Hospital, IRCCS Rome Italy; ^7^ Oxford Medical Genetics Laboratories, the Churchill Hospital Oxford UK; ^8^ Department of Pediatrics, Institute of Clinical Sciences University of Gothenburg, Queen Silvia Children's Hospital, Sahlgrenska University Hospital Gothenburg Sweden; ^9^ Center for Human Genetics, Department of Genetics and Genome Sciences University Hospitals Cleveland Medical Center, Case Western Reserve University School of Medicine Cleveland Ohio USA; ^10^ Neurological Institute, Department of Neurology University Hospitals Cleveland Medical Center, Case Western Reserve University School of Medicine Cleveland Ohio USA; ^11^ Department of Pediatrics University Hospitals Cleveland Medical Center, Case Western Reserve University School of Medicine Cleveland Ohio USA; ^12^ The Freya Foundation Bristol UK; ^13^ Department of Pediatrics, Division of Medical Genetics Stanford University School of Medicine and Lucille Packard Children's Hospital Palo Alto California USA; ^14^ Department of Pediatrics, Division of Human Genetics Mitochondrial Medicine Frontier Program, Children's Hospital of Philadelphia Philadelphia Pennsylvania USA; ^15^ Department of Pediatrics University of Pennsylvania Perelman School of Medicine Philadelphia Pennsylvania USA; ^16^ Center for Computational Genomic Medicine, Children's Hospital of Philadelphia Philadelphia Pennsylvania USA; ^17^ Section of Clinical Genetics and Metabolism, Department of Pediatrics University of Colorado School of Medicine Aurora Colorado USA; ^18^ Nutrition Services, Stanford University School of Medicine and Lucille Packard Children's Hospital Palo Alto USA; ^19^ Division of Genetic and Genomic Medicine Nationwide Children's Hospital Columbus Ohio USA; ^20^ Department of Pediatrics The Ohio State University Columbus Ohio USA; ^21^ Department of Nutrition and Dietetics Bristol Royal Hospital for Children Bristol UK; ^22^ Reference Center for Mitochondrial Disorders (CARAMMEL) and Reference Center for Inborn Errors of Metabolism, Department of Pediatrics, Necker‐Enfants‐Malades Hospital, Assistance Publique‐Hôpitaux de Paris, University of Paris‐Cité, MetabERN Paris France; ^23^ INSERM UMRS 1163, Institut Imagine Paris France; ^24^ Department of Dietetics Great Ormond Street Hospital London UK; ^25^ Division of Endocrinology, Diabetes and Metabolism, Departments of Medicine and Biochemistry and Molecular Biology, College of Medicine University of Florida Gainesville Florida USA; ^26^ The Elizabeth Watt PDCD Research Fund Centennial Colorado USA; ^27^ University Children's Hospital, Expertise Centre for Mitochondrial Diseases (Mitohaus), Paracelsus Private Medical University (PMU) Salzburg Austria; ^28^ UPMC Children's Hospital of Pittsburgh Pittsburgh Pennsylvania USA; ^29^ Division of Genetic and Genomic Medicine, Department of Pediatrics University of Pittsburgh Pittsburgh Pennsylvania USA

**Keywords:** clinical guideline, ketogenic diet, outcomes, pyruvate dehydrogenase complex deficiency, pyruvate dehydrogenase deficiency, treatments

## Abstract

Primary pyruvate dehydrogenase complex deficiency (PDCD) comprises a group of monogenic disorders caused by pathogenic variants in genes encoding subunits of, or regulatory components affecting, the pyruvate dehydrogenase complex. The clinical phenotype spans a broad continuum, ranging from early onset congenital lactic acidosis to infantile or childhood onset global developmental delay with epilepsy, through to more attenuated adult‐onset neurological presentations. Reports from patients and advocacy groups indicate substantial variability in clinical management across both emergency and outpatient settings and between centres internationally. This variability underscores the need for systematic evaluation of the evidence base and the development of harmonised, consensus‐driven clinical guidelines to standardise care and improve outcomes. An international consortium of experts from Europe and North America, including metabolic physicians, neurologists, dietitians, geneticists and patient representatives, was convened. The group undertook a structured review of the literature and developed guideline statements addressing disease classification, recognition, diagnostic evaluation, dietary and non‐dietary management, surveillance for complications, genetic counselling and transition to adult services. Each recommendation was assigned a GRADE rating reflecting strength and quality of evidence. Consensus was achieved using a Delphi methodology. In total, 199 recommendations reached consensus and constitute the core of these guidelines. These recommendations provide a framework for consistent, high‐quality, multidisciplinary care. The process also identified key evidence gaps, highlighting priorities for future research and the ongoing need to develop effective disease‐modifying therapies.

## Graphical abstract

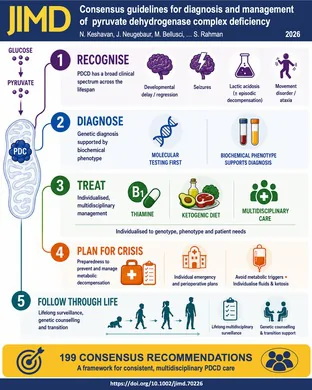

## 1 Introduction

The pyruvate dehydrogenase complex (PDC) catalyses the conversion of pyruvate to acetyl‐CoA in mitochondria. This key reaction links glycolysis with the tricarboxylic acid (TCA) cycle and electron transport chain. PDC is fundamentally important in the brain where most energy generated is derived from glucose. Elsewhere, PDC plays a central role in regulating the balance of substrate utilisation and in the nucleus provides acetyl‐CoA for acetylation reactions.

PDC is composed of multiple copies of three catalytic components, pyruvate dehydrogenase (E1), dihydrolipoamide acetyltransferase (E2), dihydrolipoamide dehydrogenase (E3) and one structural component, E3 binding protein (E3BP). Each catalytic component has a cofactor: thiamine diphosphate (ThDP) for E1, lipoic acid for E2 and flavin adenine dinucleotide (FAD) for E3 [1, 2, 3].

The first step of the reaction cycle is the oxidative decarboxylation of pyruvate by E1 with ThDP. CO_2_ is released and the remaining 2‐carbon unit is transferred via an ethylidene‐ThDP intermediate to the E2 enzyme where it reductively acetylates a lipoamide cofactor. E2 then catalyses the transfer of the acetyl group to CoA, leaving the thiols of the lipoamide cofactor in their reduced state. These are subsequently re‐oxidised by E3 with the reduction of its FAD cofactor. The cycle is completed by the re‐oxidation of the FAD with NAD^+^, yielding acetyl‐CoA and NADH as the final products (Figure 1A).

**FIGURE 1:**
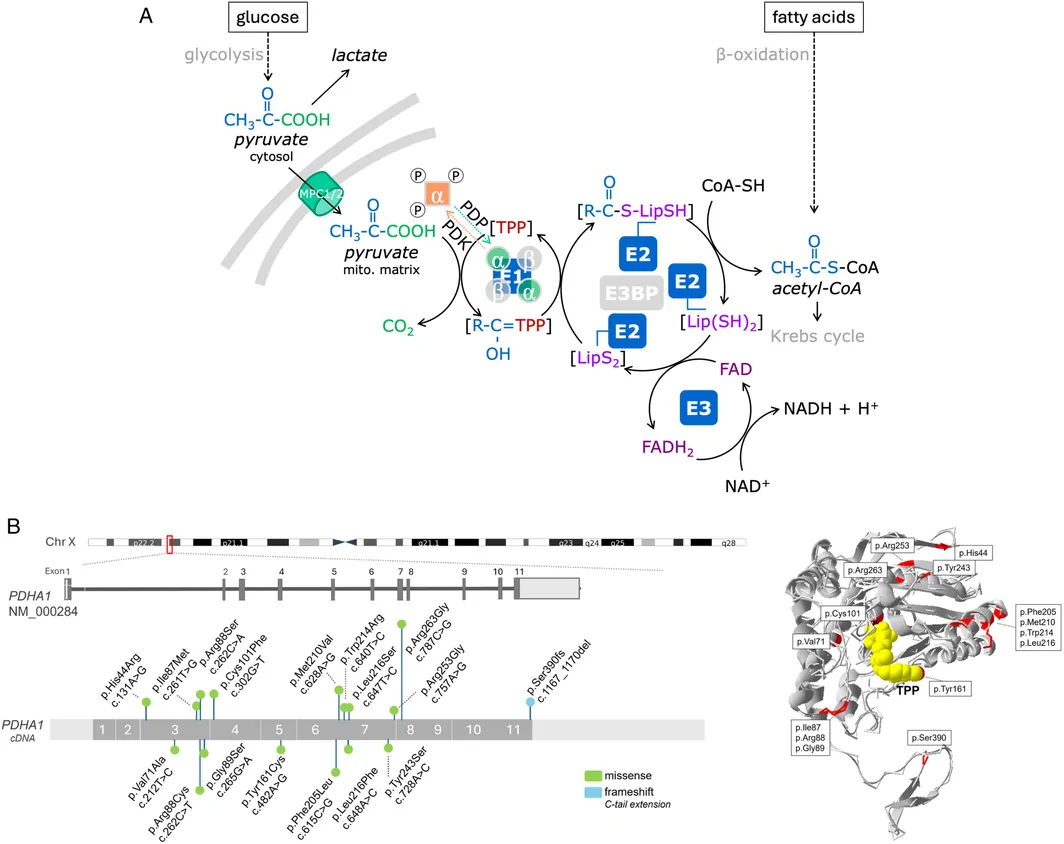
(A) PDCD complex biochemistry and related pathways. CoA: coenzyme A, CO_2_: carbon dioxide, E1, E2, E3, E3BP: subunits of PDCD, FAD: flavin adenine dinucleotide, Lip: lipoic acid, NAD: nicotinamide adenine dinucleotide, PDK: pyruvate dehydrogenase kinase, PDP: pyruvate dehydrogenase phosphatase, TPP: thiamine pyrophosphate. (B) Thiamine responsive pathogenic variants of *PDHA1*. The chromosomal location of *PDHA1* gene, its 11 exons and cDNA are shown in the left panel. The crystal structure of the E1A subunit is shown on the right. The loci of thiamine responsive pathogenic variants are shown along the cDNA and in red on the crystal structure.

E3 is not unique to PDC but is shared with other α‐ketoacid dehydrogenase complexes, α‐ketoglutarate dehydrogenase complex and branched chain α‐ketoacid dehydrogenase complex, which also share their overall molecular architecture and reaction mechanism.

PDC activity is subject to close control in different tissues under different nutritional conditions and disease states [1, 2, 3]. Its activity is regulated by specific kinases (PDKs) and phosphatases (PDPs) which phosphorylate and dephosphorylate the E1α subunit resulting in inactivation and reactivation of the complex, respectively. While pyruvate inhibits PDK thereby activating PDC, increased NADH/NAD^+^ and acetyl‐CoA/CoA ratios activate PDK thereby acting as negative regulators. As a result of these mechanisms, the overall activity of the complex is responsive to dynamic alterations in the relative concentrations of key intermediates and metabolic circumstances [1, 2, 3].

Primary PDC deficiency (PDCD) can result from pathogenic variants in any one of the genes encoding subunits of the complex; however, most cases are due to defects in *PDHA1*, the X‐linked gene encoding the E1α subunit. There are several reports of pathogenic variants in *PDHX*, which encodes E3BP [4, 5, 6, 7, 8], but only a few cases of defects in *PDHB* [9, 10, 11, 12] and *DLAT* [13, 14], the genes encoding E1β and E2, respectively. Pathogenic variants in *DLD*, the gene encoding E3, are found relatively frequently in some ethnic groups [15, 16] and result in a combined deficiency of all α‐ketoacid dehydrogenase complexes containing this subunit, frequently presenting with clinical features that differ from those seen in other forms of PDCD. Pathogenic variants in *PDP1* are also reported to cause PDCD [17, 18, 19].

Currently there are no international guidelines for diagnosing and managing patients with PDCD. Feedback from patient advocacy groups has highlighted that this has resulted in inequitable care, confusion among non‐experts and risk of clinical error surrounding specific aspects of care, especially emergency and perioperative management and use of ketogenic diet. This initiative aims to close this gap by formulating a comprehensive guideline which will delineate best practice, minimise potential ambiguities and standardise clinical care.

## 2 Methods

### 2.1 Scope of Guideline

This guideline pertains to primary PDCD caused by pathogenic variants in the following genes: *PDHA1*, *PDHB*, *DLAT*, *PDHX* and *PDP1*. DLD deficiency will not be considered in the current guidelines as it differs substantially from the other primary deficiencies both in terms of its clinical and biochemical presentation and its management. Secondary deficiencies of PDCD, for example, those related to biosynthesis of lipoic acid and ThDP, will also not be considered.

### 2.2 Guideline Development

This guideline has been developed by combining consensus opinion of experts in the field and evidence obtained through appraisal of the published literature. The Delphi method was used to develop consensus guidelines for recognition, diagnosis and management of primary PDCD, together with Grading of Recommendations, Assessment, Development and Evaluation (GRADE) methodology to evaluate the strength of recommendations as detailed by the International Guideline Harmonization group (https://www.ighg.org/international‐guideline‐harmonization‐group/methods/grading‐system/). Before and during meetings, guideline group members received training in standardised literature evaluation according to GRADE methodology to assess the quality of evidence and determine the strength of recommendation for each statement. The guideline working group consisted of 19 physicians (metabolic specialists, biochemical geneticists, neurologists, biochemists), three metabolic dietitians and five patient advocacy group representatives. At the outset, the patient groups were asked to formulate questions and queries that they would like the guideline to address. They were also involved throughout the guideline’s development in a consultative capacity, including input into conceptualisation and wording of guideline statements, but did not participate in voting during the Delphi process. Among the 21 voting members, the gender distribution was 10 females and 11 males. Geographic representation was equally balanced between the United States and Europe.

Eight working groups were formed, each tasked with defining key questions in one of the following subjects: (1) biochemical and biophysical characteristics of PDC; (2) clinical features of PDCD; (3) diagnostic approaches; (4) management approaches; (5) dietary management; (6) surveillance for disease complications; (7) genetic counselling; and (8) transition to adult services. Monthly virtual meetings and additional interim subgroup meetings occurred over a 12 month period from 14 October 2024.

### 2.3 Literature Review

A literature review on PDCD was performed in January 2025 in the following databases: PubMed, Google Scholar, Cochrane Library and ClinicalTrials.gov. Publications were excluded if they were not relevant to the predefined key questions or were not written in English. A total of 377 peer reviewed publications were identified as relevant to the topic. Details of search terms and the PRISMA flow chart are provided in the Supporting Information.

### 2.4 Consensus Procedure

The initial voting process was carried out remotely between September and November 2025 online using a survey‐based platform (Qualtrics, https://login.qualtrics.com) set up at UCL, UK. Voting was conducted using a structured Delphi methodology.

Each guideline statement and its corresponding GRADE score was voted upon using a 5‐point Likert scale (1: absolutely disagree; 2: disagree; 3: neutral; 4: agree; 5: absolutely agree). ‘Strong consensus’ was pre‐defined as > 70% of scores being ≥ 4 or ≤ 2 with a mean score ≥ 4 or ≤ 2. If either condition was met, then the statement was deemed to have reached ‘consensus’. If neither parameter was met, the statement was considered to lack consensus. Survey data were compiled and analysed to determine the level of consensus for each statement.

A face‐to‐face meeting of the whole guideline group took place in Cambridge, UK on 12–14 November 2025. All guideline statements not reaching consensus, and those with suggested amendments or having at least one member indicate absolute disagreement, were discussed, including level of evidence and optimal wording of the statements. Changes to the statements were made and voting repeated using Mentimeter (https://menti.com) during the face‐to‐face guideline group meeting in November 2025 and online using Qualtrics in January–February 2026.

## 3 Recommendations

Raw data from voting within the Delphi process and the degree of consensus for each statement are included in the Supporting Information.

### 3.1 Clinical Features of PDCD

To aid clinical recognition of PDCD, a literature search aimed to rigorously evidence the clinical spectrum of disease, categorised by age of onset (prenatal, neonatal, infantile/early childhood, older children, adolescents and adults). These features are summarised in guideline statements **C1–C28** in Table 1.

**TABLE 1:** Guideline statements regarding clinical features of PDCD.

No.	Guideline statement	GRADE level of evidence	References
	**Prenatal presentation**		
C1	Prenatal disease is most frequently observed in females with *PDHA1*‐related disease but has also been reported in males with *PDHA1*‐related disease, as well as in individuals with *PDHB*‐ and *PDHX*‐related forms of PDCD.	**B**	[9, 20, 21, 22, 23, 24, 25, 26, 27, 28, 29]
C2	Typical neurodevelopmental and brain abnormalities in prenatal onset PDCD include microcephaly, migration abnormalities such as pachygyria, polymicrogyria, periventricular nodular heterotopias, as well as corpus callosum hypoplasia or agenesis. Additional findings often include cerebellar and brainstem hypoplasia, for example, hypoplastic dentate nuclei and pyramidal tracts. Rarely, midbrain malformations resulting in aqueductal stenosis and hydrocephalus have been reported.	**B**	[22, 23, 30, 31]
C3	Corresponding neuroimaging features of prenatal onset PDCD include decreased supra‐ and infratentorial grey and white matter volumes, ventriculomegaly, periventricular cysts and ventricular membranes.	**B**	[22, 23, 30, 31]
C4	Histopathological or anatomical lesions observed in PDCD include asymmetric leukomalacia, reactive gliosis, large germinolytic pseudocysts and basal ganglia lesions.	**B**	[22, 23, 30, 31]
C5	Typical features of craniofacial dysmorphism in PDCD include microcephaly, a narrow head with frontal bossing, a broad nasal bridge, an upturned nose with flared nostrils, a long and smooth philtrum, thin lips, low‐set ears and retrognathism.	**B**	[20, 24, 25, 26, 27, 28, 32]
C6	Dysmorphic features involving other body regions occur in PDCD and may include small hands and feet, rhizomelic limb shortening and single palmar creases. Visceral malformations are generally absent in individuals with PDCD.	**B**	[20, 24, 25, 26, 27, 28, 32]
C7	Intrauterine growth restriction and low birth weight are frequently observed in PDCD, affecting approximately one‐third of cases.	**B**	[21, 28, 30, 33]
	**Neonatal presentation**		
C8	Neonatal presentation is at least as frequent in females with pathogenic *PDHA1* variants as in males, and affected females appear to have a lower overall mortality rate compared with affected males.	**B**	[7, 9, 17, 18, 19, 21, 33, 34, 35, 36, 37, 38, 39, 40]
C9	Neonatal presentations of PDCD have also been reported in individuals with pathogenic variants in *PDHB*, *PDHX* and *PDP1*. Neonatal presentations are only rarely described in individuals with pathogenic variants in *DLAT*.	**B**	[7, 9, 17, 18, 19, 21, 33, 34, 35, 36, 37, 38, 39, 40]
C10	The most frequent clinical manifestations of PDCD in the neonatal period are hypotonia and lactic acidosis or acidaemia, often accompanied by respiratory distress in the context of acute acid–base imbalance. Other common features include encephalopathy characterised by lethargy, sometimes accompanied by epileptic seizures, as well as feeding difficulties and failure to thrive.	**B**	[7, 8, 9, 17, 18, 19, 20, 21, 25, 26, 33, 34, 35, 36, 37, 38, 39, 40, 41, 42, 43, 44]
C11	A majority of affected neonates with PDCD exhibit evidence of prenatal disease onset.	**B**	[7, 8, 9, 20, 21, 25, 26, 33, 34, 35, 36, 41, 42, 43]
C12	Infrequently reported findings in PDCD presenting in the neonatal period include leukoencephalopathy with cerebral white matter oedema, muscular hypertonia or dystonia, and abnormal ocular movements.	**B**	[21, 33, 34]
	**Infantile/early childhood presentation**		
C13	Hypotonia and developmental delay are the most common clinical findings (≥ 50%) in both males and females presenting in infancy or early childhood with pathogenic variants in *PDHA1* or other genes associated with PDCD.	**B**	[24, 26, 33]
C14	Other common clinical findings in early onset PDCD include feeding difficulties, microcephaly, seizures, ataxia, dystonia, brainstem dysfunction, peripheral neuropathy and dysmorphic features.	**B**	[24, 26, 33, 45]
C15	Other clinical findings in early onset PDCD include spasticity, choreoathetosis and optic atrophy.	**B**	[26]
C16	Ophthalmological disease involvement in PDCD may include refractive errors (e.g., myopia/hypermetropia/astigmatism), optic nerve dysplasia/atrophy, strabismus. Visual impairment or loss is frequent in PDCD, usually due to abnormalities of the higher visual pathways (cortical blindness).	**B**	[29]
C17	Sleep disorders and sleep–wake disturbances (such as excessive daytime sleepiness, fragmented sleep and sleep‐disordered breathing) are common in individuals with intellectual disability and have been reported even more frequently in genetic syndromes and inherited metabolic diseases including primary mitochondrial diseases.	**B**	[46, 47, 48]
C18	Clinical features are characterised by variability and a broad spectrum of severity in all genetic subtypes of PDCD.	**B**	[20, 49]
C19	Neuroimaging findings in infantile/early childhood onset PDCD include microcephaly, asymmetric ventriculomegaly, cerebral atrophy, cerebellar atrophy, polymicrogyria, corpus callosum abnormalities, periventricular pseudocysts and features in the Leigh syndrome spectrum, including basal ganglia and brainstem abnormalities.	**B**	[21, 26, 50, 51]
C20	Females with pathogenic variants in *PDHA1* frequently have significant neuroimaging findings, especially ventriculomegaly, corpus callosum abnormalities and other structural brain abnormalities.	**B**	[21, 51, 52, 53, 54]
C21	Patients with pathogenic variants in *PDHB*, *PDHX*, *DLAT* and *PDP1* may have similar neurological features to PDCD caused by pathogenic variants in *PDHA1*.	**B**	[7, 8, 13, 19, 33, 36, 37, 55]
	**Presentation in older children (~ 4–5+ years at first symptom), adolescents and adults**		
C22	Patients with PDCD may present for the first time in adolescence or adulthood with ataxia, peripheral neuropathy and dystonia, which are typical manifestations seen in patients within this age group. Other patients develop these manifestations in later life after having first presented with clinical features associated with the infantile‐ or early childhood‐onset disease phenotype.	**B**	[26, 56, 57, 58, 59, 60, 61, 62, 63, 64]
C23	While ataxia and peripheral neuropathy have been most frequently described in males with PDCD, they also appear relatively frequently in females and may be underrecognised in this population.	**B**	[7, 19, 20, 21, 26, 33, 50, 56, 57, 58, 59, 65, 66, 67, 68, 69, 70, 71, 72, 73, 74, 75, 76, 77, 78, 79, 80, 81, 82, 83, 84]
C24	Peripheral neuropathy is axonal more often than demyelinating, but can be either of these forms in PDCD.	**B**	[7, 20, 21, 26, 33, 50, 57, 58, 59, 66, 70, 76, 78, 79, 82, 83]
C25	Dystonia may be the initial presenting feature of PDCD or be superimposed on a more global early‐onset presentation.	**B**	[20, 33, 55, 57, 59, 60, 61, 66, 68, 72, 73, 74, 82, 83, 85, 86, 87, 88, 89]
C26	Dystonia and dyskinesia are frequent presentations of DLAT deficiency.	**B**	[55, 60, 72, 80, 89]
C27	Other movement disorders—such as chorea, parkinsonism and tremor—have also been described in older children and adults with PDCD, though less frequently than ataxia, neuropathy and dystonia. Patients may have more than one subtype of movement disorder.	**B**	[56, 57, 69, 73, 77, 78, 80, 83, 85, 90]
C28	An increasingly diverse spectrum of clinical features is described in PDCD that overlaps with other primary mitochondrial disorders: episodic weakness (often precipitated by carbohydrates or intercurrent illness), exercise intolerance, ptosis, stroke‐like episodes, Leigh syndrome spectrum neuroimaging findings, dysarthria, focal seizures, migraines, dementia, psychiatric symptoms, ophthalmoplegia, diplopia, loss of vision and optic neuropathy.	**B**	[20, 21, 24, 55, 57, 58, 59, 60, 61, 62, 63, 65, 66, 67, 68, 69, 70, 73, 74, 79, 80, 82, 83, 85, 86, 87, 88, 89, 91, 92, 93, 94]

Prenatal disease onset occurs frequently in PDCD, affecting approximately 50% of individuals [20, 21], identified on routine fetal ultrasound. When PDCD manifests before 26 weeks of gestation, essential fetal neurodevelopmental processes are disrupted, including neuronal proliferation, migration and organisation. In contrast, energy failure occurring later in gestation tends to result in discrete brain lesions. As a result, most individuals with prenatal‐onset PDCD present with a combination of developmental abnormalities and discrete brain lesions [9, 21, 22, 23, 24, 30, 31]. These include microcephaly, neuronal migration abnormalities such as pachygyria, polymicrogyria, periventricular nodular heterotopias, corpus callosum hypoplasia or agenesis, cerebellar and brainstem hypoplasia [22, 23, 30, 31]. Neuroimaging may demonstrate decreased brain volume, ventriculomegaly or periventricular cysts [22, 23, 30, 31]. Craniofacial dysmorphism is a frequent feature in PDCD [20, 24, 25, 26, 27, 28, 32]. The features often resemble those seen in fetal alcohol syndrome, a similarity that may be attributed to the effects of acetaldehyde, a metabolite of alcohol which is elevated in cases of alcohol exposure and is known to be a potent inhibitor of the pyruvate dehydrogenase complex (PDC) [24, 32]. These craniofacial features include microcephaly, a narrow head with frontal bossing, a broad nasal bridge, an upturned nose with flared nostrils, a long and smooth philtrum, thin lips, low‐set ears, and retrognathism [20, 24, 25, 26, 27, 28, 32]. Intrauterine growth restriction and low birth weight are frequently observed in PDCD [21, 28, 30, 33].

Neonatal clinical presentation is characterised by hypotonia, lactic acidosis, respiratory distress, encephalopathy, lethargy, seizures, feeding difficulty and failure to thrive [7, 8, 9, 17, 18, 19, 20, 21, 25, 26, 33, 34, 35, 36, 37, 38, 39, 40, 41, 42, 43, 44]. Many neonates demonstrate evidence of prenatal disease onset. Neonatal presentation is described in PDHA1, PDHB, PDHX and PDP1 deficiencies but rarely in DLAT deficiency.

Infantile/childhood disease presentation is characterised by significant variability in symptoms and disease severity. Clinical features include hypotonia, developmental delay, feeding difficulties, microcephaly, spasticity and optic atrophy [24, 26, 33, 45]. Patients may experience paroxysmal symptoms including breakthrough seizures, ataxia, stroke‐like episodes, somnolence, intermittent weakness and dystonia. Rarely, individuals present later in childhood with intermittent ataxia, paroxysmal dystonia or dyskinesia or other atypical clinical findings such as alternating hemiplegia or episodic limb paralysis.

In older children and adults, PDCD may manifest for the first time with ataxia and/or peripheral neuropathy [7, 19, 20, 21, 26, 33, 50, 56, 57, 58, 59, 65, 66, 67, 68, 69, 70, 71, 72, 73, 74, 75, 76, 77, 78, 79, 80, 81, 82, 83, 84]. Peripheral neuropathy takes various forms in PDCD, most often axonal, but may include recurrent demyelination or Guillain‐Barre‐like syndrome resembling chronic inflammatory demyelinating polyneuropathy [7, 20, 21, 26, 33, 50, 57, 58, 59, 66, 70, 76, 78, 79, 82, 83]. Other late‐onset features include dystonia, typically episodic [20, 33, 55, 57, 59, 60, 61, 66, 68, 72, 73, 74, 82, 83, 85, 86, 87, 88, 89], movement disorders such as chorea, parkinsonism and tremor [56, 57, 69, 73, 77, 78, 80, 83, 85, 90], episodic weakness (often precipitated by carbohydrates or intercurrent illness), exercise intolerance, ptosis, stroke‐like episodes, Leigh syndrome spectrum neuroimaging findings, dysarthria, focal seizures, migraines, dementia, psychiatric symptoms, ophthalmoplegia, diplopia, loss of vision and optic neuropathy [20, 21, 24, 55, 57, 58, 59, 60, 61, 62, 63, 65, 66, 67, 68, 69, 70, 73, 74, 79, 80, 82, 83, 85, 86, 87, 88, 89, 91, 92, 93, 94].

### 3.2 Diagnostic Testing

Guideline statements **D1–D11** refer to diagnostic testing (Table 2A).

**TABLE 2A:** Guideline statements pertaining to diagnosis of PDCD.

No.	Guideline statement	GRADE strength of recommendation	GRADE level of evidence	References
D1	When PDCD is initially suspected, blood lactate and pyruvate should be measured and CSF sampling should be considered.	**I**	**C**	[95, 96]
D2	Plasma amino acids and urine organic acids should be measured when PDCD is suspected.	**I**	**C**	[97, 98, 99]
D3	Carbohydrate loading tests are not necessary and should not be done.	**I**	**C**	Expert opinion
D4	Regardless of whether an institution has a quantitative measure of pyruvate, molecular testing is the diagnostic test of choice.	**IIa**	**C**	[33]
D5	If financially feasible, molecular testing should be pursued in parallel with biochemical testing because the former provides a definitive genetic diagnosis while the latter may provide complementary essential information.	**IIa**	**C**	[100]
D6	In a patient with clinically suspected PDCD (with or without suggestive biochemical findings), a molecular‐first or molecular‐in‐parallel approach should be undertaken, with the selection of clinical exome or genome sequencing as the test of choice versus a targeted gene panel, dependent on the institutional financial availability.	**IIa**	**C**	[100]
D7	Prognostication should not be based on genotype alone.	**I**	**B**	[50]
D8	Normal PDC enzyme activity, particularly in skeletal muscle and lymphocytes, especially in a female, does not exclude PDCD.	**I**	**B**	[4, 95, 100]
D9	Decreased PDC enzyme activity may be a secondary phenomenon and should not be used as the sole basis for clinical management.	**I**	**B**	[4, 95, 100, 101, 102]
D10	While X‐inactivation studies are not routinely indicated for prognostication in female patients with *PDHA1*‐related PDCD, they may provide valuable insights in cases with atypical clinical features or when sequencing results show a mosaic pattern of wild‐type and mutant alleles.	**IIb**	**C**	[42, 84, 103, 104]
D11	Somatic mosaicism in *PDHA1* is reported in males and females with heterogeneous PDCD phenotypes.	**I**	**C**	[104, 105, 106, 107]

#### 3.2.1 Biochemical Testing

We recommend measuring lactate and pyruvate and calculating the lactate/pyruvate ratio which is typically low to normal (10–20), but can be > 20 in patients who are acutely ill, or be spuriously elevated in blood following difficult venepuncture or because of pyruvate degradation during suboptimal sample handling [97, 108]. Lactate and pyruvate elevation is more frequent in CSF than in blood [95, 96]. Some patients with PDCD may have normal blood and CSF lactate and pyruvate. We recommend that plasma amino acids and urine organic acids be measured; these tests are more sensitive post‐prandially after a carbohydrate‐containing meal and less sensitive after fasting. Alanine may be elevated (reflecting transamination of elevated pyruvate) but ratios of alanine to other amino acids, such as alanine:leucine (≥ 4.0), proline:leucine (≥ 3.0), (alanine+proline):leucine (≥ 6.5), (alanine+proline):(leucine+lysine) (≥ 2.5) and alanine:lysine (≥ 3.0) may be elevated even when alanine is normal [97, 98, 99]. Specific amino acid ratios are highly sensitive but not specific for PDCD. Urine organic acids in PDCD typically show elevations of lactate and pyruvate, but non‐specific elevations in some organic acids and tricarboxylic acid intermediates may also be seen, depending on the genetic aetiology of PDCD [97, 98, 99].

Carbohydrate loading tests are not recommended. However, in healthcare contexts where genetic testing is either too costly or unavailable, a carbohydrate loading test could be considered, although the rigour needed to obtain and interpret a meaningful result is challenging.

#### 3.2.2 Enzymatic Testing

PDC enzyme activity is typically measured by quantifying ^14^CO_2_ release from [1‐^14^C]‐pyruvate in cell or tissue homogenates [109, 110]. Cultured skin fibroblasts are the patient cells of choice for assessing PDC enzymatic activity, but lymphocytes and skeletal muscle may be used. PDC activity assays in cultured skin fibroblasts are highly sensitive for detecting *PDHA1*‐related disease, with sensitivities of 97% in females and 91% in males, outperforming activity assays in lymphocytes and muscle [100]. However, normal enzyme activity can be seen in female patients, related to skewed X‐inactivation. Lymphocyte‐ and muscle‐based testing lack sensitivity: 36% and 58%, respectively, for identifying known PDC‐deficient females with pathogenic *PDHA1* mutations [100].

Protein studies, such as immunoblotting or proteomics, may identify missing components, but often this is only available on a research basis [4].

The working group carefully considered the role of measuring PDC activity in diagnostic testing and concluded after much discussion that it should not be routinely recommended. The working group acknowledged that most centres in the United States and Europe do not offer PDC enzymology presently and historically very few laboratories in the world are able to carry out the test reliably to the required accredited standard. The assay is also no longer available in several countries owing to limited availability of the labelled substrate. Furthermore, the test is not 100% specific since low PDC activity may be seen in secondary causes of PDCD, which are outside the scope of this guideline. For the diagnosis of PDP1 deficiency, PDP1 enzymology is challenging to perform, requires specific assay conditions and is complex to interpret owing to the presence of isoenzymes. Molecular testing is therefore the test of choice to confirm primary PDCD. Enzymatic testing is an adjuvant methodology that can be used if genetic testing is inconclusive.

#### 3.2.3 Assessment of Thiamine Responsiveness in *PDHA1* Disease

In the past, PDC enzyme activity was used to assess thiamine responsiveness in vitro by identifying a dose‐dependent response of PDC activity to ThDP at both low and high ThDP concentrations. The working group discussed that this testing is no longer available to an accredited standard in most centres. Furthermore, it was noted that in vitro evidence of thiamine responsiveness frequently does not correlate with clinical thiamine responsiveness. Clinicians in the working group agreed they would not withhold thiamine from patients while they are awaiting results of in vitro responsiveness testing or indeed in patients for which in vitro testing suggests non‐responsiveness to thiamine. Molecular genetic testing can identify variants known to be thiamine‐responsive including those that affect residues needed for thiamine binding in E1α (Figure 1B), but other variants may also show thiamine responsiveness. PDC enzyme activity may be less sensitive in patients with thiamine‐responsive variants if performed in a thiamine‐rich medium, a limitation that should be considered when evaluating variants of uncertain significance.

#### 3.2.4 Genetic Testing

Molecular genetic diagnostic testing is the test of choice regardless of availability or results of biochemical investigations. However, genetic and biochemical testing are often undertaken in parallel, and we support either a genetics‐in‐parallel approach or a genetics‐first approach. The choice of molecular testing—targeted genetic panel or broader genomic approach—depends on clinical suspicion, financial availability and local turnaround times. Variants in *PDHA1* have variable expressivity, creating challenges in predicting phenotype from genotype. Prognostication should not be based on genotype alone [50].

#### 3.2.5 X Inactivation and Somatic Mosaicism Studies

X inactivation studies are not usually needed in diagnostic testing but may be useful in interpreting sequencing data that indicates mosaicism between wild‐type and mutated alleles. X inactivation studies usually use peripheral tissue (fibroblasts or lymphocytes) and may not correlate with other tissues such as the brain, the predominantly affected organ in PDCD. Cultured cells from females with *PDHA1*‐related PDCD past passage p10 may develop selection bias towards cells that express only the wild‐type allele. X inactivation patterns ≥ 70:30 bias in favour of the wild‐type allele are associated with normal enzyme activity [42, 84, 103]. Conventional X inactivation assays can detect skewing but typically cannot determine which X chromosome (mutant or wild‐type) is preferentially active without further molecular data [42, 84, 103].

### 3.3 Genetic Counselling

Guideline statements **G1–G4** refer to genetic counselling (Table 2B). *PDHA1‐*related PDCD is inherited in an X‐linked manner whereas pathogenic variants in *PDHB, DLAT, PDP1* and *PDHX* exhibit autosomal recessive inheritance. Molecular testing is important to determine the specific subtype of PDCD and for genetic counselling regarding mode of inheritance and recurrence risk in subsequent pregnancies. Mothers of children with *PDHA1* variants, the most common genetic aetiology of PDCD, should be offered genetic testing to determine whether the variant is maternally inherited or de novo. Most affected males (60%–63%) have de novo variants [57, 111] but a significant minority are maternally inherited. Most affected females (85%–95%) have de novo variants [57, 111] and a smaller minority of pathogenic variants are maternally inherited. If the proband's variant is not detected in the mother, recurrence risk is low but above the general population risk because of possible germline mosaicism.

**TABLE 2B:** Guideline statements pertaining to genetic counselling for families of patients diagnosed with PDCD.

No.	Guideline statement	GRADE strength of recommendation	GRADE level of evidence	References
G1	All patients with a clinical, biochemical or enzymatic diagnosis of PDCD should undergo testing to determine the genetic aetiology of PDCD.	**I**	**C**	[33]
G2	All families should be referred for genetic counselling once a genetic diagnosis of PDCD is made. This includes discussion regarding mode of inheritance and implications for other family members including future pregnancies.	**I**	**C**	Expert opinion
G3	Mothers of patients diagnosed with genetically confirmed *PDHA1‐*related PDCD should be offered targeted genetic testing to determine whether the pathogenic variant is maternally inherited.	**I**	**B**	[57, 111]
G4	In PDCD due to recessive mode of inheritance (*PDHB*, *DLAT*, *PDP1*, *PDHX*), parental segregation for the pathogenic variants should be confirmed by genetic testing. Preimplantation or prenatal genetic testing may be discussed with families after the causative pathogenic variants are identified.	**I**	**B**	[57, 111]

### 3.4 Management

Guideline statements about management of PDCD are subdivided into general (**M1–M19**, Table 3A), metabolic drug (**M20–M29**, Table 3B), emergency (**M30–M46**, Table 3C), perioperative (**M47–M52**, Table 3D) and dietetic management (**M53–M66**, Table 3E).

**TABLE 3A:** Guideline statements pertaining to general management of patients with PDCD.

No.	Guideline statement	GRADE strength of recommendation	GRADE level of evidence	References
M1	For optimal management, PDCD usually requires a multidisciplinary team based at a specialised centre, working closely with community care providers and other levels of care. As the disease progresses, the care plan and points of contact for each stage should be regularly updated. Clear and consistent communication should be maintained with patients and their families throughout the process.	**I**	**C**	Expert opinion
M2	The multidisciplinary team should include an inherited metabolic disease expert or a neurologist with experience in PDCD as the coordinator, together with a dietitian (for patients on KD), a neurologist, and speech, physical and occupational therapists. Neuropsychological follow‐up and educational programmes should also be part of the comprehensive care plan. The care plan and team composition should be individualised according to the patient's needs and the healthcare context.	**I**	**C**	Expert opinion
M3	When starting new drugs (e.g., oral antibiotics) in patients with PDCD, use sugar‐free formulations where possible, to minimise the risk of disrupting ketosis and thus metabolic control.	**I**	**B**	[112, 113, 114]
M4	If enteral feeds are being tolerated, continue feeding the patient (orally or via a gastric tube) their KD or usual high‐fat formula. Maintaining the KD is essential for metabolic stability in PDCD.	**I**	**B**	[115, 116, 117, 118, 119, 120]
M5	Carbohydrates may precipitate lactic acidosis in PDCD, therefore high dose glucose (> 5% dextrose) infusions should be avoided.	**I**	**B**	[112, 115, 116, 117, 118, 119, 120]
M6	For patients with PDCD who are fasting > 24 h (including surgery/anaesthesia), parenteral nutrition containing glucose (≤ 5%), lipids and amino acids should be administered.	**I**	**B**	[43, 121]
M7	PDCD is not yet a curable disease, but specific and personalised management can greatly help reduce symptoms and ease the disease burden. The specific approach depends on the patient and the specific disease (phenotype and genotype). Molecular genetic confirmation of the PDCD diagnosis is necessary to implement a personalised treatment plan.	**I**	**B**	[7, 29, 50, 72, 122]
M8	Most patients with PDCD need multiple therapies including KD, thiamine and symptomatic therapies, for example, antiseizure medications and other therapies where indicated.	**I**	**B**	[72]
M9	For seizure control, good clinical practice including general indications, contraindications, clinical monitoring and side effects for all antiseizure medications must always be kept in mind.	**I**	**C**	Expert opinion
M10	Topiramate, due to higher risk of precipitating acidosis, should not be considered a first‐line treatment for seizures in PDCD, although its use in specific cases is not absolutely contraindicated.	**I**	**B**	[123]
M11	Caution should be exercised when considering the use of propofol to treat refractory status epilepticus in PDCD due to the risk of propofol infusion syndrome.	**I**	**C**	[123]
M12	In patients with PDCD, medications that can induce acidosis should be avoided. If their use is unavoidable, they must be administered with close clinical and biochemical monitoring.	**IIa**	**C**	[123, 124, 125]
M13	In the advanced stages of the disease, or when the disease burden requires it, access to palliative care services is valuable for managing symptoms, improving quality of life, and supporting emotional and family needs.	**I**	**C**	Expert opinion
M14	It is important to remain vigilant in preventing and promptly managing respiratory infections in PDCD, with families actively involved in monitoring for warning signs.	**I**	**A**	[50, 126, 127]
M15	Patients with PDCD should have a swallowing evaluation by a specialised team if there is a clinical concern.	**I**	**A**	[50, 126, 127]
M16	Diet modifications and other compensatory techniques can help manage dysphagia, support caloric intake and potentially reduce aspiration risk.	**I**	**A**	[50, 126, 127]
M17	In patients with diagnosed dysphagia or frequent respiratory infections or aspiration pneumonia, the use of a nasogastric tube or surgical or endoscopic placement of a feeding tube should be considered promptly to prevent complications and improve nutritional status and outcomes.	**I**	**A**	[50, 126, 127]
M18	Palliative and complex care teams should be integrated early in the clinical course, to provide essential guidance, emotional support, and symptom and therapeutic management, helping families navigate the complexities of care. This proactive involvement enhances the patient's and their family's quality of life and empowers caregivers to make well‐informed decisions regarding treatment and comfort measures.	**I**	**C**	Expert opinion
M19	An advanced palliative care plan including end‐of‐life considerations should be established early, clearly outlining goals of care, anticipated complications and patient and family preferences.	**I**	**C**	Expert opinion

**TABLE 3B:** Guideline statements pertaining to metabolic drug management of patients diagnosed with PDCD.

No.	Guideline statement	GRADE strength of recommendation	GRADE level of evidence	References
M20	Thiamine supplementation 150–1000 mg/day ideally in three divided doses is recommended in patients with PDCD.	**I**	**B**	[8, 20, 51, 58, 128, 129, 130, 131, 132, 133, 134, 135, 136, 137, 138, 139, 140, 141, 142]
M21	For *PDHA1* or *PDHB* variants of unknown thiamine‐responsiveness, a dose‐dependent response of fibroblast‐based PDC activity to thiamine pyrophosphate may be considered to identify thiamine‐responsive PDCD. There are a number of known *PDHA1* variants that are thiamine‐responsive.	**I**	**B**	[8, 20, 51, 58, 128, 129, 130, 131, 132, 133, 134, 135, 136, 137, 138, 139, 140, 141, 142]
M22	Thiamine supplementation may help control paroxysmal symptoms with proper long‐term adherence, and reduce the frequency of paroxysmal episodes in PDCD.	**I**	**B**	[20, 59, 115, 117, 132]
M23	Apart from thiamine, evidence supporting the use of other vitamins and cofactors in PDCD remains limited.	**N/A**	**C**	[143, 144, 145, 146]
M24	L‐carnitine deficiency is a possible complication of ketogenic diet. L‐carnitine supplementation may be considered if low levels of free carnitine are observed in bloodspot or plasma.	**IIa**	**C**	[143, 144, 145, 146, 147]
M25	Metabolic acidosis is a possible complication of ketogenic diet. Bicarbonate, and/or citrate supplementation may be needed to treat metabolic acidosis in patients with PDCD.	**IIa**	**C**	[143, 144, 145, 146, 148]
M26	Dichloroacetate (DCA) is an investigational drug. It is an activator of the PDC and works by inhibiting pyruvate dehydrogenase kinase. It helps to reduce lactic acid levels in both plasma and CSF.	**IIa**	**C**	[149, 150, 151, 152, 153]
M27	The investigational drug DCA has been shown to be safe and well tolerated in patients with PDCD following acute intravenous or chronic oral use.	**IIa**	**B**	[149, 150, 151, 152, 153]
M28	The recommended dosing of the investigational drug DCA depends on the EGT haplotype of *GSTZ1*, distinguishing fast metabolisers (25 mg/kg/day) from slow metabolisers (12.5 mg/kg/day).	**IIa**	**B**	[150]
M29	Clinical benefits of the investigational drug DCA in patients with PDCD remain to be established, pending the results of a recently completed randomised controlled trial.	**N/A**	**C**	Expert opinion

*Note:* N/A: not applicable, no GRADE Strength of Recommendation because the statement does not contain a recommendation.

**TABLE 3C:** Guideline statements pertaining to emergency management of patients diagnosed with PDCD.

No.	Guideline statement	GRADE strength of recommendation	GRADE level of evidence	References
M30	It is essential to provide patients with PDCD an emergency management letter outlining their condition and suggested management of key complications, including special recommendations for those on KD.	**I**	**C**	[112, 115, 116, 154, 155, 156]
M31	Acute metabolic decompensation events in PDCD may be precipitated by periods of metabolic stress including fasting, insufficient food intake, inappropriately high carbohydrate intake, vomiting, diarrhoea, intense physical exercise, fever, intercurrent illness, anaesthesia and surgery.	**N/A**	**B**	[26, 50, 72, 119, 154, 157, 158, 159, 160]
M32	The most typical biochemical findings of metabolic decompensation events in PDCD include worsening of lactate (above usual baseline), metabolic acidosis and excessive ketosis.	**N/A**	**B**	[7, 26, 72, 94, 158, 159]
M33	Symptoms of metabolic decompensation in PDCD include worsening of neurological status (confusion, lethargy, seizure, focal neurological signs), laboured or rapid breathing, fatigue, difficulty ambulating, fast heart rate, headache and vomiting.	**N/A**	**B**	[26, 51, 68, 72, 94, 158, 159, 160]
M34	When acute metabolic decompensation is suspected clinically, peripheral venous catheterisation should be undertaken and blood tests obtained before starting IV fluids.	**I**	**C**	Expert opinion
M35	The metabolic tests include: blood gas, glucose, electrolytes, bicarbonate, liver and kidney function, lactate, ketones. The general blood and urine tests include CBC, CRP, blood cultures if febrile, urinalysis.	**I**	**C**	Expert opinion
M36	During acute metabolic decompensation events in PDCD, medical therapy should be concurrently instituted to treat any underlying intercurrent illness.	**I**	**C**	Expert opinion
M37	In cases of elevated lactate above the individual's baseline, particularly during acute illness, secondary causes such as severe infection, sepsis, or cardiorespiratory instability must be promptly excluded.	**I**	**A**	[161]
M38	Where hydration is required, aim to use fluids containing the lowest possible dextrose level, while maintaining blood glucose levels ≥ 2.5 mmol/L or ≥ 45 mg/dL. Extreme vigilance should be used providing dextrose containing fluids whether or not the patient is on KD.	**I**	**C**	Expert opinion
M39	During management of metabolic decompensation, use age‐appropriate saline‐based IV fluids but if there are concerns regarding hypoglycaemia or prolonged IV use, consider adding low dextrose fluids (e.g., to a final concentration of 2.5% dextrose).	**I**	**C**	Expert opinion
M40	Ringer lactate (also known as lactated Ringer's solution or Hartmann's solution) is contraindicated in PDCD.	**I**	**B**	[156]
M41	If there is metabolic acidosis (pH < 7.20) and/or bicarbonate < 10 mmol/L and/or base deficit >10 mmol/L, then IV bicarbonate (or citrate) could be considered.	**I**	**C**	Expert opinion
M42	If there is ketosis > 5 mmol/L in acute metabolic decompensation in PDCD, IV glucose should be avoided, and patients should be hydrated using normal saline/electrolyte‐balanced crystalloids to decrease ketone levels.	**I**	**C**	Expert opinion
M43	During periods of fasting for elective procedures or during acute illness when feeds are not being tolerated, metabolic monitoring of glucose and ketones and blood gas should be undertaken 4 hourly.	**I**	**C**	Expert opinion
M44	Patients on KD can typically tolerate low blood sugar levels, symptomatic hypoglycaemia is more likely when glucose levels are < 2.5 mmol/L. IV glucose should be administered when hypoglycaemia occurs.	**I**	**C**	Expert opinion
M45	Severe acute decompensations of PDCD require management in an intensive care unit.	**I**	**B**	[72]
M46	EEG and MRI are valuable tools for diagnosing and managing neurological manifestations in severe acute decompensations of PDCD.	**I**	**B**	[72]

*Note:* N/A: not applicable, no GRADE Strength of Recommendation because the statement does not contain a recommendation.

Abbreviations: CBC, complete blood count; CRP, C‐reactive protein.

**TABLE 3D:** Guideline statements pertaining to perioperative management of patients diagnosed with PDCD.

No.	GRADE strength of recommendation	GRADE strength of recommendation	GRADE level of evidence	References
M47	Slow titration and dose adjustment of volatile and parenteral anaesthetics are recommended in PDCD to minimise adverse effects.	**I**	**C**	[157, 162, 163, 164, 165, 166, 167]
M48	Prolonged use of propofol during maintenance anaesthesia may elevate the risk of lactic acidosis in PDCD.	**I**	**B**	[157, 162, 163, 164, 165, 166, 167]
M49	Neuromuscular blocking agents should be avoided in individuals with muscle disease or used only under strict monitoring if absolutely necessary.	**I**	**B**	[168]
M50	Stressors, such as changes in body temperature, catabolism and drugs that exacerbate lactic acidosis (including lactate‐containing agents), should be avoided perioperatively in PDCD.	**I**	**B**	[157, 162, 163, 164, 165, 166, 167]
M51	Close monitoring of acid–base balance, glucose, ketone bodies and lactate levels is essential during the perioperative period in patients with PDCD.	**I**	**B**	[157, 162, 163, 164, 165, 166, 167]
M52	Fasting and the use of intravenous glucose during the perioperative period in prolonged anaesthesia should be individualised in patients with PDCD, particularly for those on ketogenic diet.	**I**	**C**	Expert opinion

**TABLE 3E:** Guideline statements pertaining to dietetic management of patients diagnosed with PDCD.

No.	Guideline statement	GRADE strength of recommendation	GRADE level of evidence	References
M53	Based on the available evidence we recommend the use of ketogenic diet (KD) in the management of PDCD.	**I**	**C**	Expert opinion
M54	We recommend that KD should be used in the management of other genetic forms of primary PDCD (*PDHB*, *DLAT*, *PDHX* and *PDP1*) given the shared underlying pathophysiology. although the evidence about KD in PDCD largely pertains to patients with *PDHA1* variants.	**I**	**C**	Expert opinion
M55	The classical KD is recommended for patients with PDCD but a modified KD could be considered in individual patients, for example, with milder disease, later onset or difficulties with tolerance.	**I**	**C**	Expert opinion
M56	KD can be delivered via various modes, including oral, enteral and parenteral routes. The route should be determined by the patient's overall clinical condition.	**IIa**	**C**	Expert opinion
M57	In PDCD there is no specific target ketone level. Target blood ketone levels are individualised to each patient and are at the level at which they are clinically stable.	N/A	**C**	Expert opinion
M58	KD should be started as soon as feasibly possible following confirmed genetic diagnosis of primary PDCD, regardless of age.	**I**	**C**	Expert opinion
M59	KD remains the primary treatment for PDCD and should not be delayed in favour of a thiamine trial, particularly in patients presenting with seizures and severe lactic acidosis.	**I**	**C**	Expert opinion
M60	KD often leads to a decrease in lactate levels in PDCD, although the response may vary.	N/A	**C**	Expert opinion
M61	There is some evidence in the published literature of improvement in quality of sleep after implementation of KD	N/A	**C**	Expert opinion
M62	There is some evidence in the published literature of progression with social interaction after implementation of KD	N/A	**C**	Expert opinion
M63	There is some evidence in the published literature of improvement in motor development after implementation of KD.	N/A	**C**	Expert opinion
M64	For patients with seizures, there is evidence in the published literature that KD is effective in seizure reduction.	N/A	**C**	Expert opinion
M65	In patients with PDCD, following initiation of KD, there may be improvement in seizures, ataxia, Leigh syndrome spectrum decompensation episodes, movement disorders, motor development and social interaction. There may be minor improvement in sleep but no normalisation in sleep behaviour. There may be a decrease in hospital admissions.	N/A	**C**	Expert opinion
M66	KD may help control paroxysmal symptoms during the acute phase and, with proper long‐term adherence, reduce the frequency of paroxysmal episodes in PDCD.	N/A	**B**	[115, 117]

*Note:* N/A: not applicable, no GRADE Strength of Recommendation because the statement does not contain a recommendation.

#### 3.4.1 General Management

See Table 3A for general management recommendations (**M1–M19**). Patients should be managed within the context of a multidisciplinary team which combines the expertise of a metabolic disease expert or neurologist, dietitian for patients on a ketogenic diet and other allied healthcare professionals and community services depending on individual needs. Depending on their individual problems, patients with PDCD will need multiple therapies to manage symptoms, but care should be taken when commencing new medications to ensure that they do not contain carbohydrates in patients on a ketogenic diet to prevent disruption of ketosis [112, 113, 114].

We recommend that existing published guidelines [123] are followed for management of seizures in patients with PDCD. However, topiramate should be avoided as first‐line treatment since it may cause acidosis, and propofol should be used cautiously in the management of status epilepticus owing to risk of propofol infusion syndrome due to inhibition of fatty acid oxidation [123]. Patients and families should be provided access to palliative care services for advice on managing symptoms, and for family support, especially at advanced disease stages.

#### 3.4.2 Metabolic Drug Management

Guideline statements regarding metabolic drug management are listed in Table 3B. We recommend thiamine supplementation (150–1000 mg/day in three divided doses) in patients with PDCD. Thiamine is reported to improve lactic acidosis, muscle weakness, paroxysmal episodes, movement disorders and may help stabilise the disease, regardless of whether there is a thiamine‐responsive genetic variant [8, 20, 51, 58, 128, 129, 130, 131, 132, 133, 134, 135, 136, 137, 138, 139, 140, 141, 142]. Patients with thiamine‐responsive PDCD variants (Figure 1B) often show paroxysmal symptoms and a marked clinical improvement after thiamine treatment, although the supporting evidence remains limited [8, 20, 51, 58, 128, 129, 130, 131, 132, 133, 134, 135, 136, 137, 138, 139, 140, 141, 142]. In other patients, thiamine may still be beneficial, considering its potential effect in stabilising the PDC enzyme.

Patients with free carnitine deficiency secondary to ketogenic diet therapy should receive supplementation [143, 144, 145, 146, 147]. Bicarbonate or citrate may be used in management of metabolic acidosis [143, 144, 145, 146, 148]. Dicholoroacetate is an investigational drug with known safety and tolerability which activates PDC by inhibiting PDK, thereby lowering lactate levels [149, 150, 151, 152, 153]. A recently completed clinical trial aimed to assess efficacy of DCA in management of PDCD (NCT02616484), with results yet to be reported.

#### 3.4.3 Emergency Management

Patients with PDCD may experience acute metabolic decompensations during intercurrent illnesses or other metabolic stressors such as fasting, insufficient food intake, inappropriately high carbohydrate intake, anaesthesia and surgery, even after initiation of disease‐specific treatment [112, 115, 116, 154, 155, 156]. Patients should receive an emergency management letter to be taken to the emergency department during acute illnesses. Key biochemical abnormalities include metabolic acidosis, elevated lactate and ketones above the patient's usual baseline.

Figure 2 summarises the acute management of patients with PDCD during metabolic decompensation, including necessary blood investigations. Where IV hydration is needed, we recommend saline‐based fluids with no or low dextrose content since high glucose delivery will exacerbate metabolic acidosis in PDCD and negate therapeutic ketosis in patients on KD. Lactate‐containing fluids are contraindicated. Dextrose‐containing fluids may be used with caution in specific situations, including hypoglycaemia < 2.5 mmol (45 mg/dL) and in prolonged IV fluid use, with regular blood glucose and ketone monitoring. Patients with metabolic acidosis (pH < 7.20 and/or bicarbonate < 10 mmol/L and/or base deficit >10 mmol/L) who are acutely unwell should receive IV or enteral bicarbonate/citrate supplementation.

**FIGURE 2:**
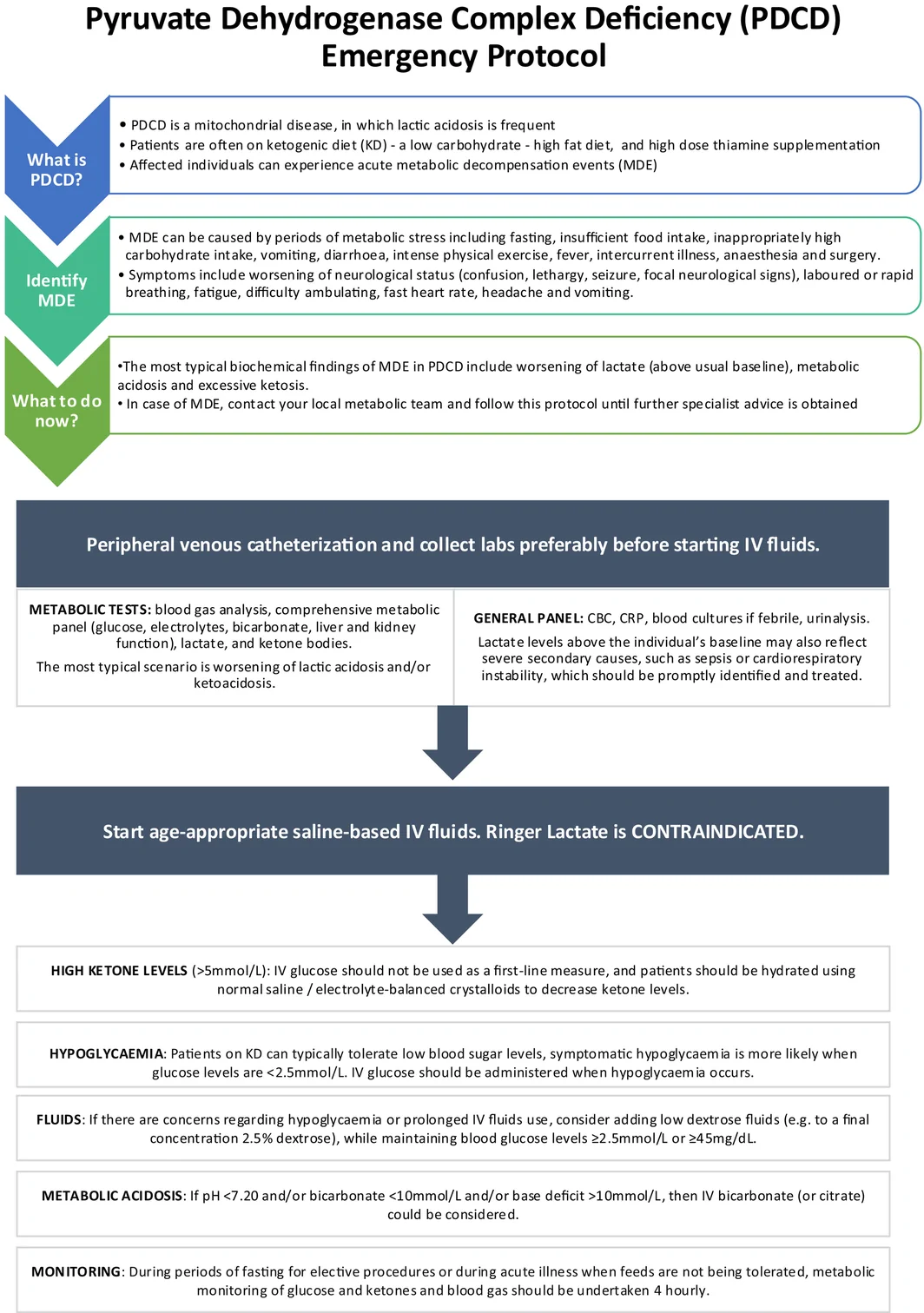
PDCD emergency protocol detailing the acute clinical presentation of PDCD, initial investigations, intravenous fluid use and monitoring. CBC: complete blood count, CRP: C‐reactive protein, KD: ketogenic diet, MDE: metabolic decompensation event.

Patients with hyperketosis > 5 mmol/L in the context of acute metabolic decompensation should be managed with IV hydration using normal saline or electrolyte‐balanced crystalloids that lack dextrose to decrease ketone levels. Once adequate hydration has been achieved, careful use of low dextrose infusion may be needed if hyperketosis persists.

In all cases, the underlying medical cause for deterioration should be investigated and treated without delay. Patients presenting with severe acute decompensation and/or acute neurological manifestations, including but not limited to Leigh syndrome spectrum lesions, leukoencephalopathy, dystonia, ataxia and epilepsy, should be admitted to an intensive care unit.

#### 3.4.4 Perioperative Management

Table 3D lists guidance statements about perioperative management. Patients with PDCD are at increased risk of anaesthesia‐related complications. Anaesthesia can exacerbate respiratory symptoms and trigger respiratory failure, requiring careful monitoring throughout the procedure [157, 162, 163, 164, 165, 166, 167]. We recommend slow titration of all anaesthetics, avoidance of neuromuscular blockade in PDCD patients with muscle involvement and advise against prolonged use of propofol which may cause lactic acidosis and interfere with ketogenesis [157, 162, 163, 164, 165, 166, 167]. Duration of preoperative fasting should be minimised, and regular perioperative monitoring of acid–base balance, glucose, ketone bodies and lactate is essential. Perioperative IV fluid considerations should be as described above for emergency management. Patients not receiving enteral feeds for more than 24 h should be commenced on parenteral nutrition and be restarted on their usual enteral feed as soon as possible, especially if on a ketogenic diet.

#### 3.4.5 Dietetic Management

Ketogenic diet (KD) was first reported to be beneficial in PDCD in 1976 [69]. KD shifts the body's primary energy source from carbohydrates to fats, promoting the production of ketone bodies, which can enter the TCA cycle and serve as an alternative fuel [116]. Ketone bodies can cross the blood–brain barrier, providing the brain with an alternative energy source to glucose. Currently, no formal guidelines exist for the use of KD in PDCD, and research is limited due to the rarity of the condition.

The KD is characterised by a high fat content, controlled protein intake to support growth, and restricted carbohydrate consumption. The goal is to induce and maintain a state of ketosis, whereby ketone bodies become the predominant energy substrate [169]. In some of the literature, the term KD was used to describe diets that were low in carbohydrate, but with high protein content (up to 30% energy). These do not align with current definitions of KD. The high protein content means protein will be directed through gluconeogenesis and glycolysis to produce pyruvate and consequently lactate; these diets will not drive the production of ketones.

There are four main types of KD, as follows.

##### 3.4.5.1 Classical Ketogenic Diet

The classical KD is based on long‐chain triglycerides and is calculated using a ratio of grams of fat to grams of combined protein and carbohydrate [169]. Commonly used ratios include 4:1 and 3:1. Protein is provided at levels sufficient to support growth and development, while carbohydrate intake is strictly limited to promote ketosis [170].

##### 3.4.5.2 Medium Chain Triglyceride Ketogenic Diet

The medium chain triglyceride (MCT) KD modifies the classical approach by incorporating MCTs as the primary fat source [171]. MCTs are more efficient at generating ketone bodies compared to long‐chain triglycerides (LCTs), allowing for greater flexibility in protein and carbohydrate intake, which can improve the diet's palatability [170]. Originally formulated with 60% MCT, the diet has since been adjusted to 30% to reduce gastrointestinal side effects associated with high MCT intake [171]. The typical macronutrient distribution is: 30%–60% MCT, 15%–45% LCT, 10% protein and 15% carbohydrate [172].

##### 3.4.5.3 Modified Ketogenic Diet

Due to the restrictive nature of classical and MCT KDs, adherence can be challenging, particularly for older children. The Modified Ketogenic Diet (MKD) offers a more flexible alternative, with a fat to protein+carbohydrate ratio of approximately 1:1. It has demonstrated efficacy in managing epilepsy in children [173]. The MKD allows for increased protein and carbohydrate intake, making it more appealing and easier to implement in daily meal planning [112].

##### 3.4.5.4 Low Glycaemic Index Diet

The Low Glycaemic Index Diet (LGID) was developed following observations that children on the KD maintained stable blood glucose levels [174]. This stability was hypothesised to contribute to the therapeutic effects of the KD [112]. The LGID permits carbohydrate intake of 40–60 g per day, provided the carbohydrates have a glycaemic index below 50 [112].

##### 3.4.5.5 Side Effects

Side effects of KD include renal calculi, hypercholesterolaemia, gastrointestinal symptoms (e.g., vomiting, constipation and diarrhoea), and poor growth, particularly in children on more restrictive versions of the diet. These potential adverse effects highlight the importance of close clinical monitoring and multidisciplinary support when initiating and maintaining KD [112].

##### 3.4.5.6 Outcomes

The evidence base for outcomes of KD management of PDCD is limited to small, descriptive cohorts or single case reports and uncontrolled observational studies [14, 43, 51, 56, 69, 112, 115, 117, 118, 154, 175, 176, 177, 178, 179, 180, 181]. Assessments of outcomes in the literature were largely subjective rather than objectively measured, reflecting a low level of evidence and highlighting the need for more rigorous controlled studies. The lack of controlled studies makes it difficult to attribute outcomes to KD rather than the natural disease course within advances in general clinical management [176]. Outcomes reported in the literature include improved biochemistry (with mixed reports regarding lactate lowering effects), neurocognitive development including social interaction and communication, gross and fine motor development, sleep, movement disorder, ataxia, seizures and reversal of brain lesions detected on MRI. There is some evidence that KD may help in decreasing the severity and frequency of paroxysmal neurological episodes.

Recommendations for use of KD in PDCD are summarised in Table 3E. We recommend that KD should be used in the management of primary PDCD caused by *PDHA1*, *PDHB, DLAT, PDHX and PDP1* variants, given the shared underlying pathophysiology. KD should be commenced as soon as possible after confirming a genetic diagnosis of PDCD and excluding disorders in which KD is contraindicated (e.g., fatty acid oxidation disorders, DLD and ECHS1 deficiency). The choice of KD, formulation and route of administration should be tailored to the individual's clinical condition, and their and the family's ability to adhere to the diet. Target ketone levels are individualised to the patient, based on symptom control.

### 3.5 Disease Monitoring and Surveillance

Guideline recommendations **S1–S78** about disease monitoring in PDCD are listed in Table 4A and summarised in Table 4B. They are grouped into the following domains: multidisciplinary care, laboratory tests, gastroenterology, psychomotor delay/intellectual disability/cognitive impairment, neuropsychiatric and mental health, seizures, disorders of movement and tone (ataxia, dystonia, choreoathetosis, parkinsonism, spasticity, tremor), peripheral neuropathy, neuroimaging, cardiology, respiratory function, orthopaedics/bone health, kidney/renal function, dentistry/drooling, hearing, ophthalmology, sleep and pain. For many of these domains, the recommendations are not specific to PDCD, but feedback from patient advocates emphasised the need to provide guidance in these areas for PDCD.

**TABLE 4A:** Guideline statements pertaining to disease monitoring/surveillance in patients with PDCD.

No.	Guideline statement	GRADE strength of recommendation	GRADE level of evidence	References
	**Multidisciplinary care**			
S1	All patients with highly suspected or confirmed PDCD disease should be referred to a centre of expertise.	**I**	**B**	[182, 183, 184, 185, 186]
S2	A rare disease specialist (inherited metabolic disease specialist, geneticist, paediatric/adult neurologist) with clinical experience in PDCD supported by a local multidisciplinary team (including at minimum a dietitian with experience in ketogenic diet), should lead the patient's care.	**I**	**C**	Expert opinion
S3	A care and assessment plan should be individualised to the patient and healthcare context, integrated at the beginning of care and be regularly updated according to the patient's clinical course and needs	**I**	**B**	[187, 188]
S4	Psychosocial support should be offered (where available) to families after diagnosis.	**I**	**C**	Expert opinion
S5	Families should be informed about local and/or international patient organisations that offer support for PDCD.	**I**	**C**	Expert opinion
S6	Families should be informed of clinical trials for PDCD locally and internationally and provided links to trial information, for example, clinicaltrials.gov in United States, euclinicaltrials.eu in the EU or equivalent in other regions.	**I**	**C**	Expert opinion
S7	Implementation of regular physical, speech/swallow, occupational and other supportive therapies should be recommended and tailored to patients' specific needs to prevent complications and to preserve optimal functioning and quality of life.	**I**	**A**	[189]
	**Laboratory monitoring**			
S8	Regular laboratory assessments are recommended to enable early recognition of complications caused by the disease or its therapy, as well as to assess nutritional status	**I**	**C**	Expert opinion
S9	Although they should be adjusted to the individual patient's needs, we recommend the following laboratory tests and intervals. For neonates and infants depending on their metabolic stability, either every 3 months or at least every 6 months. For older children (< 12 yo), we recommend an interval of every 6–12 months. For teenagers and adults, we recommend an interval of once per year	**I**	**C**	Expert opinion
S10	Core Parameters for laboratory monitoring include the following: Full blood count with differential, electrolytes, transaminases and liver function tests, kidney function (urea, creatinine, cystatin c and cystatin based eGFR if possible in patients with renal stones, impaired kidney function and/or low muscle mass), blood lactate, full blood gas, plasma/serum amino acids, ferritin, micronutrients	**IIa**	**C**	Expert opinion
S11	Additional parameters for monitoring (in particular for patients on ketogenic diet) include blood beta‐hydroxybutyrate, pancreatic function with lipase (and amylase), lipid panel (triglycerides and cholesterol), plasma free and total carnitine and a full dipstick urinalysis.	**IIa**	**C**	[112, 176, 178, 190, 191, 192, 193]
	**Gastroenterology**			
S12	Nutritional status of patients with PDCD should be closely monitored. Anthropometrics (weight, height, head circumference, growth velocity, in infants and children) should be assessed at each follow up.	**I**	**C**	Expert opinion
S13	Assessment of nutritional status should not only rely on weight and height measurements but should include regular monitoring of other physical signs of undernutrition (e.g., muscle wasting, loss of body fat) and measurement of micronutrients (e.g., protein/albumin, plasma/serum amino acids, iron status, 25‐hydroxy vitamin D, parathyroid hormone, vitamin B12, folate, calcium, phosphate, magnesium, selenium, zinc) at least annually.	**IIa**	**B**	[194, 195, 196]
S14	Careful attention should be paid to ensure adequate hydration particularly those on KD as PDCD patients may have a reduced ability to communicate thirst and may experience decreased fluid intake due to swallowing difficulties and losses due to drooling.	**I**	**B**	[175, 176, 192]
S15	Hydration needs should be adjusted for patients on a ketogenic diet.	**I**	**B**	[112, 115, 175, 176, 190, 191, 192, 193]
S16	A percutaneous enteral gastrostomy (PEG) feeding tube is recommended for individuals unable to meet nutritional requirements, those at high risk of aspiration or with confirmed silent aspiration on video fluoroscopy or those experiencing recurrent aspiration pneumonia. Discussion should be started early regarding insertion of PEG tube as this is the optimal mode of delivery of enteral feeding.	**I**	**B**	[195, 196]
S17	Gastrointestinal dysmotility is a common issue in PDCD and may present as delayed gastric emptying (gastroparesis) manifesting with symptoms such as nausea, reflux, vomiting, abdominal bloating or discomfort after meals. Clinical suspicion should prompt referral to a gastroenterologist for further investigation (e.g., gastric emptying studies).	**I**	**C**	[197]
S18	Constipation is frequent, often secondary to reduced mobility and underlying gastrointestinal dysmotility. It can also be exacerbated by dietary changes (e.g., ketogenic diet), medications, or low fluid intake. Constipation should be assessed routinely and managed early with standard of care (e.g., fibre supplement, osmotic agents).	**I**	**C**	Expert opinion
S19	Gastro‐oesophageal reflux disease (GERD/GORD) is very common in PDCD patients with neurological impairments and can contribute to feeding difficulties, vomiting or respiratory symptoms due to aspiration. GERD should be suspected in individuals with frequent regurgitation, feeding refusal, chronic cough or unexplained irritability. Management may include pharmacological therapy, dietary modifications and surgical intervention.	**I**	**B**	[198, 199, 200, 201]
S20	A trial of antireflux therapy (e.g., proton pump inhibitors) should be considered in patients with suspected GERD or unexplained signs of pain.	**I**	**B**	[196, 201, 202, 203]
	**Psychomotor delay/intellectual disability/cognitive impairment**			
S21	Neurodevelopmental and cognitive status should be closely monitored at each follow‐up since individuals with PDCD are at risk of developmental disabilities and neurological regression.	**I**	**B**	[8, 26, 29, 50, 204, 205, 206]
S22	A standardised neuropsychological or psychoeducational assessment appropriate for age is indicated at the time of diagnosis for individuals with PDCD. Repeated testing is recommended based on suspected changes in functioning, disease progression and for monitoring efficacy of treatment.	**I**	**C**	Expert opinion
S23	Developmental regression at any age warrants a thorough evaluation for a potentially treatable disease complication as well as an assessment for disease progression and evaluation of the ongoing treatment regimen/ketogenic diet. Testing may need to include repeat neuroimaging and an electroencephalogram where there are concerns that there may be subclinical seizures.	**IIa**	**B**	[20, 24, 50, 207, 208, 209]
S24	Individuals with PDCD and speech impairment should be evaluated by a speech and language therapist for the possible need of speech and augmentative communication therapy.	**I**	**C**	Expert opinion
S25	Individuals with PDCD and global developmental delay/intellectual disability (ID) require special educational services and individualised education plans in school. Referrals to a developmental specialist and appropriate therapist, and for community and school support services, should be considered. Anticipatory guidance should be provided to parents, siblings and the family.	**I**	**B**	[189, 205, 210, 211]
	**Neuropsychiatric and mental health**			
S26	Individuals with PDCD are at increased risk for neuropsychiatric and mental health issues. Depending on the age, the patient should be screened for autism and attention deficit disorder with or without hyperactivity, and/or psychiatric symptoms, for example, depression and anxiety. Referral for psychiatric evaluation is recommended in positively screened patients. Individual and family support as well as behavioural and pharmacological therapy should be initiated according to standard practices.	**I**	**B**	[29, 62, 115, 204, 205, 206, 208, 212]
	**Seizures**			
S27	Pharmacological treatment of seizures in PDCD should follow general guidelines for epilepsy treatment similar to other mitochondrial disorders, according to the guidelines published by the InterERN Mitochondrial Working Group.	**I**	**B**	[123]
S28	Good clinical practice must always be kept in mind for all antiseizure medications, including general indications, contraindications, clinical monitoring and side effects.	**I**	**C**	Expert opinion
S29	Individuals with PDCD who develop seizures should be referred to a specialist in neurology for advice about epilepsy management.	**I**	**C**	Expert opinion
S30	Seizures in PDCD are predominantly generalised but may be focal in onset and are treated with antiseizure medications based on standard practice according to seizure semiology and EEG findings.	**I**	**B**	[28, 29, 50, 115, 123, 207, 209]
S31	KD is an efficient treatment option for the underlying disease and has been shown to be an effective treatment for epileptic seizures in PDCD.	**I**	**B**	[115, 175, 176]
S32	Epileptic spasms are frequently seen in infants with PDCD. We recommend an EEG awake and asleep for early detection of hypsarrhythmia or other EEG abnormalities, with referral to a paediatric neurologist if indicated.	**I**	**B**	[26, 45]
S33	If infantile spasms or hypsarrhythmia is detected in an infant with PDCD then the first line management is KD rather than steroids.	**I**	**C**	Expert opinion
S34	No ASM is absolutely contraindicated in PDCD. Caution should be taken for ASMs that are known to be associated with metabolic acidosis (e.g., topiramate, zonisamide)	**I**	**C**	Expert opinion
S35	An acute management plan for seizure deterioration and status epilepticus is needed.	**I**	**C**	Expert opinion
S36	The management plan for worsening seizures should include review of the KD formulation, together with a specialist dietitian.	**I**	**C**	Expert opinion
S37	Any antiseizure medications should be of a carbohydrate‐free formulation, particularly in individuals with PDCD on KD	**I**	**B**	[112, 175, 176]
	**Disorders of movement and tone (ataxia, dystonia, choreoathetosis, parkinsonism, spasticity, tremor)**			
S38	Neurological assessment for disorders of movement and tone is needed at each outpatient visit.	**I**	**B**	[29, 55, 60, 68, 74, 86, 213, 214, 215]
S39	Sudden onset of a new disorder of movement or tone should lead to an evaluation of disease progression, treatment failure and potential secondary causes. Baseline laboratory investigations and neuroimaging are recommended.	**I**	**C**	Expert opinion
S40	Management of movement and tone disorders in PDCD is similar to patients with movement disorders of other causes. Medical, procedural and surgical treatments and review of KD should be considered. Treatment can improve quality of life.	**I**	**C**	Expert opinion
S41	Caution should be used with medications that alter tone (such as baclofen, benzodiazepines, anticholinergic medication such as trihexyphenidyl) as they can selectively worsen level of consciousness, decrease muscle strength and respiratory effort, and impact GI motility and bladder function.	**I**	**C**	Expert opinion
S42	Individuals with PDCD should receive physical and occupational therapy input commensurate to the severity of their motor function deficits, to maximise gross and fine motor function including mobility, prevent contractures, joint subluxations/dislocations and alleviate discomfort and pain.	**I**	**B**	[189, 216, 217]
	**Peripheral neuropathy**			
S43	With genotype‐based dosing of DCA, there is no need to undertake additional nerve conduction study (NCS) in PDCD patients commenced on DCA, unless peripheral neuropathy is suspected clinically.	**I**	**C**	Expert opinion
S44	An NCS (together with an electromyogram) may be considered if clinical symptoms/signs are suggestive of peripheral nerve dysfunction or in case of unexplained complaints (e.g., pain, if more common causes have been excluded). The nerve conduction study should include both upper and lower extremities with evaluation of both sensory and motor nerves.	**IIb**	**C**	[57, 138, 218, 219, 220, 221, 222, 223]
S45	Treatment of peripheral neuropathy in PDCD is symptomatic and follows standard of care established for other neuropathies.	**I**	**C**	Expert opinion
	**Neuroimaging**			
S46	Neuroimaging with MRI including diffusion‐weighted imaging (and ideally with spectroscopy) should be performed at the time of diagnosis. Repeated imaging should be considered if new neurological symptoms occur or progression/deterioration of symptoms is noted.	**IIa**	**C**	[51, 224, 225, 226]
	**Cardiology**			
S47	In patients with PDCD, a baseline echocardiogram and electrocardiogram should be considered and repeat cardiac assessment should be guided by a cardiologist. Patients with postural abnormalities should also receive echocardiographic monitoring. Cardiac examinations should be guided by the specific needs or symptoms of the patient, particularly in relation to the potential cardiovascular effects of medications received.	**IIb**	**C**	Expert opinion
	**Respiratory function**			
S48	Respiratory compromise in PDCD may result from a combination of hypotonia, impaired central respiratory control, bulbar dysfunction, reduced mobility and posture abnormalities like scoliosis, leading to ineffective airway clearance, hypoventilation, sleep‐disordered breathing and increased risk of aspiration pneumonia.	**I**	**B**	[29, 50, 227, 228, 229]
S49	Baseline and ongoing respiratory evaluation should be part of routine care, especially in patients with hypotonia, scoliosis, feeding difficulties and/or recurrent respiratory infections.	**I**	**B**	[189, 211, 230]
S50	Assessment may include clinical exam, pulse oximetry, capnography or overnight oximetry/polysomnography if there are concerns about sleep‐disordered breathing or nocturnal hypoventilation. Referral to a respiratory/pulmonary specialist is recommended if abnormalities are detected or recurrent respiratory infections occur.	**I**	**C**	Expert opinion
S51	Preventive strategies include vaccinations (e.g., influenza, pneumococcal), appropriate management of feeding to reduce risk of aspiration, chest physiotherapy and secretion management including mechanical cough‐assist devices as needed.	**I**	**C**	Expert opinion
S52	Non‐invasive ventilation may be indicated for patients with sleep‐disordered breathing or (nocturnal) hypoventilation.	**I**	**C**	Expert opinion
S53	Invasive ventilation (via tracheostomy) may be necessary in patients with recurrent aspiration pneumonia, respiratory insufficiency and/or recurrent central apnoea. Risks and benefits should be discussed early and thoroughly with families before such invasive ventilation intervention is commenced.	**IIb**	**C**	Expert opinion
S54	Decisions should be integrated into a comprehensive palliative care plan, including consideration of ceilings of care and Do Not Resuscitate orders.	**I**	**C**	Expert opinion
	**Orthopaedics/Bone Health**			
S55	At diagnosis and in routine follow‐up, clinical examination should include assessment of spinal abnormalities including scoliosis, kyphosis/lordosis, joint subluxation (in particular the hips due to hip dysplasia), talipes equinovarus deformity of the feet.	**I**	**B**	[209, 230, 231, 232, 233]
S56	Symptoms including pain, changes to mobility and posture should be ascertained in order to assess the impact of these clinical examination findings.	**I**	**C**	Expert opinion
S57	Referral to orthopaedics for consideration of surgical options should be made following identification of bone and joint abnormalities throughout life, utilising plain film radiographs as a first line; additional imaging may be indicated following this, as advised by the orthopaedic team.	**I**	**B**	[189, 211, 233, 234]
S58	Referral to physiotherapist, occupational therapist, podiatrist and wheelchair service where appropriate should also be initiated.	**I**	**B**	[189, 211, 233, 234]
S59	Non‐surgical options including (but not limited to) ankle orthoses, spinal braces, mobility aids and surgical options including (but not limited to) femoral or acetabular osteotomies, Achilles and other tendon release surgery, spinal surgery for scoliosis, should be discussed with the family by the orthopaedic team, if relevant for the individual patient's clinical problems.	**I**	**C**	Expert opinion
S60	Surgical decision making should consider multidisciplinary opinions to weigh potential benefits of surgery against general, specific and anaesthetic risks. All patients having surgery should have a perioperative plan which should be agreed by the lead clinical team (inherited metabolic disease/neurology/genetics as appropriate)	**I**	**C**	Expert opinion
S61	Owing to the risk of osteoporosis due to long‐term immobilisation in many patients, biochemical assessment of bone health including bone profile, parathyroid hormone and 25‐hydroxy vitamin D should be undertaken. Bone densitometry studies (DEXA) should be considered if there is a history of fragility (atraumatic) fractures. Referral to a specialist in bone metabolism should be initiated following the identification of a fragility fracture for consideration of bone protection therapy.	**I**	**C**	Expert opinion
	**Kidney/Renal function**			
S62	Ketogenic diet increases the risk of kidney stone formation, with potential complications such as obstructive uropathy, acute and/or chronic kidney disease. The estimated incidence of kidney stones in patients on ketogenic diets is approximately 5.9% but may be even higher in patients with PDCD due to underlying metabolic acidosis. Reported stone types include uric acid stones (49%), calcium oxalate or calcium phosphate stones (36%), or mixed uric‐acid and calcium‐based stones.	**N/A**	**B**	[190]
S63	Preventive and monitoring strategies to reduce the risk of kidney stone formation in patients with PDCD, especially if on ketogenic diet include: Adequate hydration—to maintain dilute urine and reduce crystallisation risk Potassium citrate supplementation can alkalinise the urine and decrease calcium stone formation by binding urinary calcium and reducing urinary citrate depletion. Regular urine analysis: detection of haematuria, crystalluria, monitor hydration Ultrasound imaging: may be indicated periodically in high‐risk patients or in those with unexplained pain or urinary symptoms as guided by a nephrologist/urologist.	**IIa**	**C**	Expert opinion
S64	Renal tubular acidosis (RTA) is a recognised manifestation of primary mitochondrial disease and has been reported in individual cases with PDCD. While rare, RTA should be considered in cases of metabolic acidosis that appear disproportionate to lactate levels and are not explained by other common causes such as ketosis.	**I**	**C**	[235]
	**Dentistry/Drooling**			
S65	Excessive drooling/sialorrhoea is common in many patients with PDCD and can significantly impact quality of life. If present, families should be advised about avoiding saliva‐stimulating substances/drinks.	**I**	**B**	[29]
S66	There is no contraindication for regular antisialorrhoeic therapy. Thus, as with other patients experiencing sialorrhoea, anticholinergics should be considered as first‐line treatment, provided that they are a sugar‐free formulation.	**I**	**B**	Expert opinion
S67	Patients with PDCD often present with abnormal dental status, which may be associated with microcephaly, dysmorphic features, orofacial hypotonia and/or feeding difficulties. Common issues include malocclusion, enamel hypoplasia, delayed eruption/impaction and increased risk of dental caries. Early and proactive specialist dental care is essential, as well as support for oral hygiene at home and preventive interventions (e.g., fluoride treatments).	**I**	**C**	Expert opinion
	**Hearing**			
S68	Sensorineural hearing loss is well described in PDCD. Hyperacusis has also been reported. Concerns regarding a deterioration in hearing should be elicited at routine follow‐up appointments. All patients with speech delay should be referred for a baseline hearing test.	**I**	**B**	[29]
S69	At diagnosis, all patients should have an up‐to‐date formal age and developmentally appropriate hearing assessment. This may include (but is not limited to): pure tone audiometry, play or visual reinforcement audiometry, tympanometry, auditory brainstem responses and/or otoacoustic emissions.	**I**	**C**	Expert opinion
S70	Consideration of interventions such as hearing aids, cochlear implants, ear defenders and regularity of follow‐up should be guided by the audiology team.	**I**	**C**	Expert opinion
	**Ophthalmology**			
S71	All patients with delayed visual or fine motor skills should be referred to an ophthalmologist with expertise in assessing patients with inherited metabolic diseases for formal eye examination. All patients should be referred at diagnosis of PDCD if they have not already been offered ophthalmological assessment. The type of ophthalmological assessment used and follow up interval is to be determined by the ophthalmologist.	**I**	**C**	Expert opinion
S72	Surgical and non‐surgical management options are to be guided by the ophthalmology team. For those who are registered blind, adjustments should be made to their living environment, care and educational needs.	**I**	**C**	Expert opinion
	**Sleep**			
S73	Sleep disturbance may substantially worsen daytime function, behaviour and caregiver burden; therefore, assessment of sleep history should be part of routine surveillance.	**I**	**C**	Expert opinion
S74	Where indicated, overnight oximetry and/or polysomnography should be performed, with referral to sleep or respiratory specialists for guidance on airway management, ventilatory support and therapies. (see respiratory function section)	**I**	**C**	Expert opinion
S75	Reversible or partially treatable contributory factors, including medication effects and timing, pain, mood or behavioural disorders, nocturnal seizures and dysphagia/aspiration should be evaluated and managed.	**I**	**C**	Expert opinion
S76	Non‐pharmacological strategies (e.g., sleep hygiene, structured sleep–wake schedules, promotion of daytime activity/physiotherapy, weighted blankets) and medications (e.g., melatonin, sedatives with caution) may help manage sleep disturbances. Medication use should be individualised, carefully weighing potential benefits against risks such as increased hypotonia and nocturnal apnoea.	**IIa**	**C**	Expert opinion
	**Pain**			
S77	Pain should be actively assessed.	**I**	**C**	Expert opinion
S78	Children with PDCD often have limited language abilities, making pain assessment challenging. Pain may arise from various sources, including osteoporosis‐related fractures due to immobility, joint sub‐/dislocations, musculoskeletal problems, for example, scoliosis, hip subluxation, increased muscle tone (including dystonia and spasticity), dental issues, urinary retention, pressure ulcers, gastrointestinal problems such as constipation and gastro‐oesophageal reflux including eosinophilic oesophagitis. Therefore, patients should be carefully evaluated to identify potential causes of pain and receive appropriate treatment.	**I**	**C**	Expert opinion

Abbreviations: ASM, antiseizure medication; DCA, dichloroacetate; DEXA, dual‐energy X‐ray absorptiometry; EEG, electroencephalogram; eGFR, estimated glomerular filtration rate; GERD/GORD, gastro‐oesophageal reflux disease; GI, gastrointestinal; ID, intellectual disability; KD, ketogenic diet; N/A, not applicable, no GRADE Strength of Recommendation because the statement does not contain a recommendation; NCS, nerve conduction study; PEG, percutaneous enteral gastrostomy, RTA, renal tubular acidosis.

**TABLE 4B:** Summary of disease monitoring/surveillance in patients with PDCD.

Monitoring domain	
Multidisciplinary care	Referral to a centre of expertise.
Laboratory tests	**Core parameters:** Full blood count with differential, electrolytes, transaminases and liver function tests, kidney function (urea, creatinine, cystatin c and cystatin based eGFR if possible in patients with renal stones, impaired kidney function and/or low muscle mass), blood lactate, full blood gas, plasma/serum amino acids, ferritin, micronutrients. **Additional parameters (for patients on KD):** Blood beta‐hydroxybutyrate, pancreatic function with lipase (and amylase), lipid panel (triglycerides and cholesterol), plasma free and total carnitine and a full dipstick urinalysis. **Interval:** Neonates/infants: every 3–6 months Older children (< 12 yo): every 6–12 months Teenagers and adults: annually
Gastroenterology	Monitor nutritional status, anthropomorphic data at each visit. Measurement of micronutrients: protein/albumin, plasma/serum amino acids, iron status, 25‐OH‐Vitamin D, parathyroid hormone, Vitamin B12, folate, calcium, phosphate, magnesium, selenium, zinc at least annually. Early identification of gastrointestinal dysmotility, constipation, reflux and referral to gastroenterology service if indicated.
Psychomotor delay/intellectual disability/cognitive impairment	Standardised neuropsychological or psychoeducational assessment at diagnosis. Neurodevelopmental and cognitive status should be monitored at each visit. Repeat standardised assessment may be used to monitor disease progression. Early identification of special education needs and referral to specialist therapists.
Neuropsychiatric and mental health	Early identification of autism, attention deficit disorder with or without hyperactivity, and/or psychiatric symptoms, for example, depression and anxiety, and referral to the appropriate service for psychiatric evaluation.
Seizures	Identification of clinical seizures warrants EEG and referral to a paediatric neurologist. An acute management plan for seizure deterioration and status epilepticus is needed. Worsening seizures should include review of the KD formulation, together with a specialist dietitian. Anti‐seizure medications should be carbohydrate‐free. Avoid drugs that worsen acidosis.
Disorders of movement and tone (ataxia, dystonia, choreoathetosis, parkinsonism, spasticity, tremor)	Neurological assessment for disorders of movement and tone is needed at each outpatient visit and. Management of these by the appropriate specialist. Early physio and occupational therapy should be initiated.
Peripheral neuropathy	Nerve conduction study and electromyogram may be considered if clinical symptoms/signs are suggestive of peripheral nerve dysfunction.
Neuroimaging	Baseline MRI brain at diagnosis. Repeat neuroimaging to be considered if new neurological symptoms occur or there is progression/deterioration of symptoms.
Cardiology	Baseline echocardiogram and electrocardiogram should be considered at diagnosis. Echocardiographic monitoring is indicated in those with postural abnormalities. Repeat cardiac assessment should be guided by a cardiologist.
Respiratory function	Clinical exam, pulse oximetry, capnography or overnight oximetry/polysomnography if there are concerns about sleep‐disordered breathing or nocturnal hypoventilation. Referral to a respiratory/pulmonary specialist is recommended if abnormalities are detected.
Orthopaedics/bone health	Early identification of spinal abnormalities including scoliosis, kyphosis/lordosis, joint subluxation (in particular the hips due to hip dysplasia), talipes equinovarus deformity of the feet. Pain should be assessed. Referral to orthopaedics team for guidance on further imaging, management and follow‐up. Referral to physiotherapist, occupational therapist, podiatrist and wheelchair service where appropriate. Biochemical assessment of bone health including bone profile, parathyroid hormone and 25‐hydroxy vitamin D should be undertaken. Consider bone densitometry studies (DEXA) if there are fragility (atraumatic) fractures. And referral to a specialist in bone metabolism.
Kidney/renal function	**For those on KD:** Regular urine analysis: detection of haematuria, crystalluria Assessment of hydration Consider potassium citrate supplementation to alkalinise the urine and decrease calcium stone formation Ultrasound imaging: may be indicated periodically in high‐risk patients or in those with unexplained pain or urinary symptoms as guided by a nephrologist/urologist
Dentistry/drooling	Early and proactive specialist dental care. Identify excessive secretions and manage with antisialorrhoeic therapy.
Hearing	At diagnosis, baseline age‐appropriate hearing assessment. Thereafter, early identification of hearing difficulties and referral to audiology service for further assessment and intervention.
Ophthalmology	At diagnosis, baseline ophthalmology examination. Thereafter, early identification of delayed visual or fine motor skills and referral to an ophthalmologist with expertise in assessing patients with inherited metabolic diseases.
Sleep	Elicit and anticipate sleep disturbances at each follow‐up visit. Consider overnight oximetry and/or polysomnography, with referral to sleep specialist.
Pain	Careful identification of pain at each follow‐up visit.

### 3.6 Transition to Adult Services

Table 5 lists recommendations concerning transition to adult services (**T1**–**T12**). Transition is not merely transfer of care from paediatric to adult services, but a process in which patients are empowered to make decisions for themselves wherever possible. There is considerable variability in cognitive function and psychosocial ability of patients with PDCD, and this should be considered at the outset of the transition process, with the aim of involving patients as much as possible in decisions, thereby promoting autonomy. The working group recognised that the transition period is associated with increased risk since care is transferred from a paediatric team that is highly knowledgeable about the patient's medical history and needs to an adult service that is comparatively unfamiliar with the individual.

**TABLE 5:** Guideline statements pertaining to transition to adult services in patients with PDCD.

No.	Guideline statement	GRADE strength of recommendation	GRADE level of evidence
**T1**	Patients reaching adulthood should ideally be followed by an adult care team, preferably by a specialist experienced in PDCD or related neurometabolic diseases (e.g., adult metabolic medicine/biochemical genetics specialist, adult neurologist, depending on local service provisions) and a dietitian familiar with managing ketogenic diet.	**I**	**C**
**T2**	A holistic approach should be taken in the management of adult patients with PDCD incorporating care by a multidisciplinary team similar to the paediatric setting.	**I**	**C**
**T3**	If a patient is receiving end of life care, continuing care within paediatric services should be considered to minimise additional stress for patients and families.	**I**	**C**
**T4**	Discussion about and planning transition should start at least 2 years prior to the anticipated transfer to adult care services.	**I**	**C**
**T5**	A transition plan with goals and emergency instructions should be developed early during the transition process and should be reviewed at least annually.	**I**	**C**
**T6**	Even though ongoing and lifelong parental or guardian support is often necessary for patients with PDCD, patients should be involved in medical decision‐making early and progressively, tailored to their individual abilities.	**I**	**C**
**T7**	Transition readiness of patient and caregivers should be evaluated and documented in the transition plan, including addressing psychosocial maturity and autonomy of the patient.	**I**	**C**
**T8**	Patients should be provided with a skills‐based education and tools to increase their autonomy depending on their individual abilities (e.g., pill reminder apps, tablet dispensers, shopping advice for ketogenic diet).	**I**	**C**
**T9**	Where feasible, patients should have the possibility of a transitional period with at least one shared follow‐up appointment involving both paediatric and adult care.	**IIa**	**C**
**T10**	Patients should not be discharged from paediatric services until they are seen by adult services.	**I**	**C**
**T11**	The clinician/service responsible for directing care during emergency department visits for metabolic emergencies must be clearly designated.	**I**	**C**
**T12**	In the early period following transition, consultation between paediatric and adult physicians is advised for acutely unwell patients. Responsibility for leading acute management is with the adult team; this should be clearly defined and communicated.	**I**	**C**

Planning transition should begin at least 2 years before the intended transfer of care to adult services. It is important that transition is undertaken specialist‐to‐specialist, ideally including a joint in‐person appointment that enables thorough handover of the patient's medical history, medications, diet plan and emergency plan to the adult team. Transition arrangements from paediatric to adult services are highly geographically and service dependent. The working group recognised that in many centres such as those in the USA where patients are managed by a specialist geneticist with expertise in metabolic disorders and a scope of practice that is not defined by age, patients with PDCD are taken care of primarily by a single specialist team throughout their lives and therefore a formalised transition process does not occur. However, it is still important to ensure that patients and families are supported in accessing adult services in the community and given advice on how to access acute medical services.

## 4 Discussion

For rare diseases, finding high‐quality evidence to support clinical decision‐making is often limited by low patient numbers, publication bias, few randomised controlled trials and insufficient detail in published literature regarding how patients have been managed. It is therefore important to draw upon the experience of clinical and scientific experts to assess the available evidence systematically. By establishing a globally recognised clinical guideline for PDCD, we intend to break down geographical boundaries, promote equitable use of healthcare resources and provide a baseline of clinical guidance that can be revised in future to keep pace with novel therapeutic discoveries and clinical trial advances.

When developing this guideline, we ensured that we included the voice of patients from conceptualisation through to finalisation of guideline statements. During the process, especially at the face‐to‐face workshop, participants were reminded of the importance of ensuring that the guideline is written in a manner that is accessible to patients, families and to both expert and non‐expert clinicians. It is anticipated that during acute illnesses, patients and families may take this clinical guideline to the emergency department, where the medical team may be unfamiliar with the management of PDCD and may need to institute immediate emergency management before being able to consult with the patient's specialist team.

The guideline is also designed to be a resource for non‐emergency management of patients reviewed in outpatient departments with a focus on holistic multidisciplinary care. Guideline statements have been written noting that they will be referred to by doctors, dietitians and other allied healthcare professionals from different paediatric and adult specialties, reflecting the multisystemic clinical presentation of PDCD.

## 5 Conclusion

A comprehensive first clinical guideline for PDCD is presented, based on appraisal of evidence and identification of international best practice. It is hoped that this guideline will facilitate disease recognition and standardise management with the aim of improving outcomes in patients with PDCD. In future, the working group will evaluate new evidence periodically and update the recommendations accordingly.

## Author Contributions

Concept: S.R., J.K.B. Biochemical and biophysical characteristics: G.B., S.B.W., S.R., J.K.B. Clinical features subgroup: N.D., A.L., G.M.E., S.D., M.B., J.K.B., S.R. Surveillance subgroup: J.N., N.K., N.D., J.K.B., S.R. Management subgroup: P.W.S., J.N., M.B., R.D.G., M.S., J.K.B., S.R. Diagnosis subgroup: E.B., J.K.B., R.D.G., M.S., S.R. Dietetic subgroup: R.L., C.N., R.S., S.R., J.K.B., S.B.W. Transition subgroup: J.N., L.D., E.W., S.B.W., S.R., N.K. Genetic counselling subgroup: R.D.G., E.B., A.L., N.K., J.K.B. Drafting of guideline statements: all authors. Drafting of manuscript: N.K., J.N., G.B., S.R. Figures: S.B.W., M.B., J.N. Data analysis and surveys: N.K. Review and editing of manuscript: all authors.

## Funding

Development of the guideline was supported by a guideline development grant from the Society for the Study of Inborn Errors of Metabolism (SSIEM) awarded to Shamima Rahman and Nandaki Keshavan and philanthropic funding awarded to Jirair K Bedoyan. Philanthropic funding was also obtained from the Freya Foundation and the Elizabeth Watt PDCD Research Fund.

## Conflicts of Interest

N.K. has received research funding from the Freya Foundation. J.N. has nothing to disclose. She received travel support and honoraria for speaking from Nutricia Metabolics, Sanofi, P.T.C. Therapeutics and Takeda Pharmaceutical. She was on the advisory board for BioMarin Healthcare. E.B. has nothing to disclose; he has served as a consultant for Orchard Therapeutics and is a Member of MetabERN PM‐MD. S.D. is the PI of a data analysis study sponsored by Saol Therapeutics and acknowledges philanthropic support from the Katie Foundation, Zachary Foundation and Gavin Foundation. L.D. and S.D. are parent members of the Freya Foundation. G.M.E. is the site‐PI for a clinical trial sponsored by Saol Therapeutics and compassionate use protocols sponsored by PTC Therapeutics and Stealth BioTherapeutics, a co‐founder of Evvia Therapeutics, a DMC member for a clinical trial sponsored by Abliva, and is on the advisory board for Thiogenesis Therapeutics. R.D.G. has participated in sponsored project research supported by Saol Therapeutics, is the Physician lead for a clinical trial sponsored by Saol Therapeutics and has accepted philanthropic funding from HopeForPDCD. K.G. is CEO of the Freya Foundation. A.N.L. is the site‐PI for clinical trials sponsored by Saol Therapeutics, Stealth BioTherapeutics, Abliva/Pharming, Tisento, Sentanyl; has consulted for UCB Pharma and Pretzel Therapeutics; is on DSMBs for the National Institutes of Health and Glycomine; is on the scientific and medical advisory board for the United Mitochondrial Disease Foundation. C.N. and R.S. have received support to attend conferences and honoraria for speaking from Nutricia and Vitaflo International Ltd. M.S. has nothing to disclose. He is a member of MetabERN PM‐MD. E.W. and S.T. are Directors of The Elizabeth Watt PDCD Research Fund. S.B.W. has nothing to disclose; she received travel support from Nutricia Metabolics. S.R. is editor‐in‐chief of the *Journal of Inherited Metabolic Disease* and has received research funding from the Freya Foundation. Several authors of this publication are members of the European Reference Network for Rare Hereditary Metabolic Disorders (MetabERN) – Project ID No 739543. G.B., R.L., N.D. and J.K.B. have nothing to disclose.

## Supporting information


**Data S1:** Supporting Information 1.

## Data Availability

Data sets regarding survey voting are available in the Supporting Information.
