# Supplementary material for: Consensus Guidelines for Diagnosis and Management of Pyruvate Dehydrogenase Complex Deficiency
Source: J Inherit Metab Dis. 2026 Oct 2;49(6):e70226. doi: 10.1002/jimd.70226 (PMC13631685; doi:10.1002/jimd.70226)
Supplement: Supplementary file 1 [file JIMD-49-0-s001.docx]

**SUPPLEMENTARY DATA**

**SEARCH TERMS**

"PDH deficiency"[tiab] OR "Pyruvate dehydrogenase deficiency"[tiab] OR "PDHA1"[tiab] OR "PDHB"[tiab] OR "DLAT"[tiab] OR "DLD" [tiab] OR "PDHX"[tiab] OR "PDP1"[tiab] OR "PDK3"[tiab] OR "pyruvate dehydrogenase complex"[MeSH Terms] OR "pyruvate dehydrogenase complex deficiency disease"[MeSH Terms] OR "pyruvate dehydrogenase complex deficiency disease"[MeSH Terms] OR "pyruvate dehydrogenase complex deficiency disease"[MeSH Terms] OR ("pdhc"[tiab] AND "deficiency diseases"[MeSH Terms]) OR "pyruvate dehydrogenase complex deficiency disease"[MeSH Terms] OR "PDCD"[tiab] AND Humans [MESH]

*Inclusion criteria: case reports, cohort studies, reviews, clinical trials with participation of PDH patients

**PRISMA FLOWCHART**

Manuscripts / Records identified from databases:

Pubmed (n = 5549)

Cochrane (n = 55)

ClinicalTrials.gov (n = 9)

Records removed *before screening*:

Records published prior 1990 (n = 920)

Duplicate records removed (n = 153)

Manuscripts / Records screened

(n = 4540)

Manuscripts / Records excluded:

Reason: only related to DLD deficiency / DLD gene

(later excluded in the guidelines process)

(n = 2263)

Studies included in review

(n = 377)

**Identification of relevant studies via databases**

**Identification**

**Screening**

**Included**

Manuscripts / Records screened and assessed for eligibility

(n = 2277)

Manuscripts / Records excluded

(n = 1900)

Reason:

language (n=9)

not human (n=592)

not disease related (n=728)

not fulfilling inclusion criteria* (n=571)

**CONSENSUS RAW DATA**

| C1 | Prenatal disease is most frequently observed in females with PDHA1-related disease but has also been reported in males with PDHA1-related disease, as well as in individuals with PDHB- and PDHX-related forms of PDCD. GRADE B |
| --- | --- |

| C2 | Typical neurodevelopmental and brain abnormalities in prenatal onset PDCD include microcephaly, migration abnormalities such as pachygyria, polymicrogyria, periventricular nodular heterotopias, as well as corpus callosum hypoplasia or agenesis. Additional findings often include cerebellar and brainstem hypoplasia, e.g. hypoplastic dentate nuclei and pyramidal tracts. Rarely, midbrain malformations resulting in aqueductal stenosis and hydrocephalus have been reported. GRADE B |
| --- | --- |

| C3 | Corresponding neuroimaging features of prenatal onset PDCD include decreased supra- and infratentorial grey and white matter volumes, ventriculomegaly, periventricular cysts, and ventricular membranes. GRADE B |
| --- | --- |

Mean score: 4.8

% score≥4: 100%

STRONG CONSENSUS

Mean score: 5

% score≥4: 100%

STRONG CONSENSUS

Mean score: 4.8

% score≥4: 94%

STRONG CONSENSUS

Mean score: 4.6

% score≥4: 94%

STRONG CONSENSUS

| C4 | Histopathological or anatomical lesions observed in PDCD include asymmetric leukomalacia, reactive gliosis, large germinolytic pseudocysts, and basal ganglia lesions. GRADE B |
| --- | --- |

| C5 | Typical features of craniofacial dysmorphism in PDCD include microcephaly, a narrow head with frontal bossing, a broad nasal bridge, an upturned nose with flared nostrils, a long and smooth philtrum, thin lips, low-set ears, and retrognathism. GRADE B |
| --- | --- |

| C6 | Dysmorphic features involving other body regions occur in PDCD and may include small hands and feet, rhizomelic limb shortening and single palmar creases. Visceral malformations are generally absent in individuals with PDCD. GRADE B |
| --- | --- |

| C7 | Intrauterine growth restriction and low birth weight are frequently observed in PDCD, affecting approximately one-third of cases. GRADE B |
| --- | --- |

Mean score: 4.5

% score≥4: 94%

STRONG CONSENSUS

Mean score: 4.7

% score≥4: 94%

STRONG CONSENSUS

Mean score: 4.8

% score≥4: 94%

STRONG CONSENSUS

| C8 | Neonatal presentation is at least as frequent in females with pathogenic *PDHA1* variants as in males, and affected females appear to have a lower overall mortality rate compared with affected males. GRADE B |
| --- | --- |

| C9 | Neonatal presentations of PDCD have also been reported in individuals with pathogenic variants in *PDHB*, *PDHX*, and *PDP1*. Neonatal presentations are only rarely described in individuals with pathogenic variants in *DLAT*. GRADE B |
| --- | --- |

| C10 | The most frequent clinical manifestations of PDCD in the neonatal period are hypotonia and lactic acidosis or acidaemia, often accompanied by respiratory distress in the context of acute acid–base imbalance. Other common features include encephalopathy characterized by lethargy, sometimes accompanied by epileptic seizures, as well as feeding difficulties and failure to thrive. GRADE B |
| --- | --- |

| C11 | A majority of affected neonates with PDCD exhibit evidence of prenatal disease onset. GRADE B |
| --- | --- |

Mean score: 4.9

% score≥4: 100%

STRONG CONSENSUS

Mean score: 4.4

% score≥4: 88%

STRONG CONSENSUS

Mean score: 4.7

% score≥4: 100%

STRONG CONSENSUS

Mean score: 4.4

% score≥4: 88%

STRONG CONSENSUS

| C12 | Infrequently reported findings in PDCD presenting in the neonatal period include leukoencephalopathy with cerebral white matter oedema, muscular hypertonia or dystonia, and abnormal ocular movements. GRADE B |
| --- | --- |

Mean score: 4.5

% score≥4: 100%

STRONG CONSENSUS

| C13 | Hypotonia and developmental delay are the most common clinical findings (≥50%) in both males and females presenting in infancy or early childhood with pathogenic variants in *PDHA1* or other genes associated with PDCD. GRADE B |
| --- | --- |

| C14 | Other common clinical findings in early onset PDCD include feeding difficulties, microcephaly, seizures, ataxia, dystonia, brainstem dysfunction, peripheral neuropathy, and dysmorphic features. GRADE B |
| --- | --- |

| C15 | Other clinical findings in early onset PDCD include spasticity, choreoathetosis, and optic atrophy. GRADE B |
| --- | --- |

Mean score: 4.9

% score≥4: 94%

STRONG CONSENSUS

Mean score: 4.9

% score≥4: 94%

STRONG CONSENSUS

Mean score: 4.8

% score≥4: 100%

STRONG CONSENSUS

| C16 | Ophthalmological disease involvement in PDCD may include refractive errors (e.g. myopia/hypermetropia/astigmatism), optic nerve dysplasia/atrophy, strabismus. Visual impairment or loss is frequent in PDCD, usually due to abnormalities of the higher visual pathways (cortical blindness). GRADE B |
| --- | --- |

Mean score: 4.7

% score≥4: 100%

STRONG CONSENSUS

| C17 | Sleep disorders and sleep-wake disturbances (such as excessive daytime sleepiness, fragmented sleep, and sleep-disordered breathing) are common in individuals with intellectual disability and have been reported even more frequently in genetic syndromes and inherited metabolic diseases including primary mitochondrial diseases. GRADE B |
| --- | --- |

| C18 | Clinical features are characterized by variability and a broad spectrum of severity in all genetic subtypes of PDCD. GRADE B |
| --- | --- |

| C19 | Neuroimaging findings in infantile/early childhood onset PDCD include microcephaly, asymmetric ventriculomegaly, cerebral atrophy, cerebellar atrophy, polymicrogyria, corpus callosum abnormalities, periventricular pseudocysts, and features in the Leigh syndrome spectrum, including basal ganglia and brainstem abnormalities. GRADE B |
| --- | --- |

Mean score: 4.8

% score≥4: 100%

STRONG CONSENSUS

Mean score: 4.6

% score≥4: 100%

STRONG CONSENSUS

Mean score: 4.4

% score≥4: 89%

STRONG CONSENSUS

| C20 | Females with pathogenic variants in *PDHA1* frequently have significant neuroimaging findings, especially ventriculomegaly, corpus callosum abnormalities, and other structural brain abnormalities. GRADE B |
| --- | --- |

Mean score: 4.8

% score≥4: 100%

STRONG CONSENSUS

Mean score: 4.4

% score≥4: 88%

STRONG CONSENSUS

Mean score: 4.7

% score≥4: 100%

STRONG CONSENSUS

| C21 | Patients with pathogenic variants in *PDHB*, *PDHX*, *DLAT*, and *PDP1* may have similar neurological features to PDCD caused by pathogenic variants in *PDHA1*. GRADE B |
| --- | --- |

| C22 | Patients with PDCD may present for the first time in adolescence or adulthood with ataxia, peripheral neuropathy and dystonia which are typical manifestations seen in patients within this age group. Other patients develop these manifestations in later life after having first presented with clinical feat  ures associated with the infantile- or early childhood-onset disease phenotype. GRADE B |
| --- | --- |

| C23 | While ataxia and peripheral neuropathy have been most frequently described in males with PDCD, they also appear relatively frequently in females and may be underrecognized in this population. GRADE B |
| --- | --- |

Mean score: 4.6

% score≥4: 100%

STRONG CONSENSUS

| C24 | Peripheral neuropathy is axonal more often than demyelinating, but can be either of these forms in PDCD. GRADE B |
| --- | --- |

Mean score: 4.6

% score≥4: 100%

STRONG CONSENSUS

| C25 | Dystonia may be the initial presenting feature of PDCD or be superimposed on a more global early-onset presentation. GRADE B |
| --- | --- |

| C26 | Dystonia and dyskinesia are frequent presentations of DLAT deficiency. GRADE B |
| --- | --- |

| C27 | Other movement disorders—such as chorea, parkinsonism, and tremor—have also been described in older children and adults with PDCD, though less frequently than ataxia, neuropathy, and dystonia. Patients may have more than one subtype of movement disorder. GRADE B |
| --- | --- |

Mean score: 4.3

% score≥4: 94%

STRONG CONSENSUS

Mean score: 4.7

% score≥4: 100%

STRONG CONSENSUS

Mean score: 4.6

% score≥4: 100%

STRONG CONSENSUS

| C28 | An increasingly diverse spectrum of clinical features is described in PDCD that overlaps with other primary mitochondrial disorders: episodic weakness (often precipitated by carbohydrates or intercurrent illness), exercise intolerance, ptosis, stroke-like episodes, Leigh syndrome spectrum neuroimaging findings, dysarthria, focal seizures, migraines, dementia, psychiatric symptoms, ophthalmoplegia, diplopia, loss of vision, and optic neuropathy. GRADE B |
| --- | --- |

Mean score: 4.6

% score≥4: 100%

STRONG CONSENSUS

| D1 | When PDCD is initially suspected, blood lactate and pyruvate should be measured and CSF sampling should be considered. GRADE CI |
| --- | --- |

Mean score: 4.5

% score≥4: 94%

STRONG CONSENSUS

**DIAGNOSIS**

| D2 | Plasma amino acids and urine organic acids should be measured when PDCD is suspected. GRADE CI |
| --- | --- |

Mean score: 4.6

% score≥4: 94%

STRONG CONSENSUS

| D3 | Carbohydrate loading tests are not necessary and should not be done. GRADE CI |
| --- | --- |

| D4 | Regardless of whether an institution has a quantitative measure of pyruvate, molecular testing is the diagnostic test of choice. GRADE CIIa |
| --- | --- |

Mean score: 5

% score≥4: 100%

STRONG CONSENSUS

Mean score: 4.6

% score≥4: 94%

STRONG CONSENSUS

| D5 | If financially feasible, molecular testing should be pursued in parallel with biochemical testing because the former provides a definitive genetic diagnosis while the latter may provide complementary essential information. GRADE CIIa |
| --- | --- |

Mean score: 4.7

% score≥4: 93%

STRONG CONSENSUS

Mean score: 4.8

% score≥4: 100%

STRONG CONSENSUS

| D6 | In a patient with clinically suspected PDCD (with or without suggestive biochemical findings), a molecular-first or molecular-in-parallel approach should be undertaken, with the selection of clinical exome or genome sequencing as the test of choice versus a targeted gene panel, dependent on the institutional financial availability. GRADE CIIa |
| --- | --- |

| D7 | Prognostication should not be based on genotype alone. GRADE BI |
| --- | --- |

Mean score: 4.5

% score≥4: 100%

STRONG CONSENSUS

| D8 | Normal PDC enzyme activity, particularly in skeletal muscle and lymphocytes, especially in a female, does not exclude PDCD. GRADE BI |
| --- | --- |

Mean score: 4.9

% score≥4: 100%

STRONG CONSENSUS

| D11 | Somatic mosaicism in *PDHA1* is reported in males and females with heterogeneous PDCD phenotypes. GRADE CI |
| --- | --- |

| D9 | Decreased PDC enzyme activity may be a secondary phenomenon and should not be used as the sole basis for clinical management. GRADE BI |
| --- | --- |

| D10 | While X-inactivation studies are not routinely indicated for prognostication in female patients with *PDHA1*-related PDCD, they may provide valuable insights in cases with atypical clinical features or when sequencing results show a mosaic pattern of wild-type and mutant alleles. GRADE CIIb |
| --- | --- |

Mean score: 4.8

% score≥4: 100%

STRONG CONSENSUS

Mean score: 4.3

% score≥4: 87%

STRONG CONSENSUS

Mean score: 4.5

% score≥4: 100%

STRONG CONSENSUS

**GENETIC COUNSELLING**

Mean score: 4.7

% score≥4: 94%

STRONG CONSENSUS

Mean score: 4.8

% score≥4: 100%

STRONG CONSENSUS

Mean score: 4.8

% score≥4: 100%

STRONG CONSENSUS

Mean score: 4.8

% score≥4: 100%

STRONG CONSENSUS

| G1 | All patients with a clinical, biochemical or enzymatic diagnosis of PDCD should undergo testing to determine the genetic aetiology of PDCD. GRADE CI |
| --- | --- |

| G2 | All families should be referred for genetic counselling once a genetic diagnosis of PDCD is made. This includes discussion regarding mode of inheritance and implications for other family members including future pregnancies. GRADE CI |
| --- | --- |

| G3 | Mothers of patients diagnosed with genetically confirmed *PDHA1-*related PDCD should be offered targeted genetic testing to determine whether the pathogenic variant is maternally inherited. GRADE BI |
| --- | --- |

| G4 | In PDCD due to recessive mode of inheritance (*PDHB*, *DLAT*, *PDP1*, *PDHX*), parental segregation for the pathogenic variants should be confirmed by genetic testing.  Preimplantation or prenatal genetic testing may be discussed with families after the causative pathogenic variants are identified. GRADE BI |
| --- | --- |

| M1 | For optimal management, PDCD usually requires a multidisciplinary team based at a specialized centre, working closely with community care providers and other levels of care. As the disease progresses, the care plan and points of contact for each stage should be regularly updated. Clear and consistent communication should be maintained with patients and their families throughout the process. GRADE CI |
| --- | --- |

Mean score: 4.8

% score≥4: 100%

STRONG CONSENSUS

Mean score: 4.9

% score≥4: 100%

STRONG CONSENSUS

**MANAGEMENT**

Mean score: 4.9

% score≥4: 100%

STRONG CONSENSUS

| M2 | The multidisciplinary team should include an inherited metabolic disease expert or a neurologist with experience in PDCD as the coordinator, together with a dietitian (for patients on KD), a neurologist, and speech, physical, and occupational therapists. Neuropsychological follow-up and educational programs should also be part of the comprehensive care plan. The care plan and team composition should be individualized according to the patient’s needs and the healthcare context. GRADE CI |
| --- | --- |

| M3 | When starting new drugs (e.g., oral antibiotics) in patients with PDCD, use sugar-free formulations where possible, to minimize the risk of disrupting ketosis and thus metabolic control. GRADE BI |
| --- | --- |

| M4 | If enteral feeds are being tolerated, continue feeding the patient (orally or via a gastric tube) their KD or usual high-fat formula. Maintaining the KD is essential for metabolic stability in PDCD. GRADE BI |
| --- | --- |

Mean score: 4.5

% score≥4: 94%

STRONG CONSENSUS

| M5 | Carbohydrates may precipitate lactic acidosis in PDCD, therefore high dose glucose (>5% dextrose) infusions should be avoided. GRADE BI |
| --- | --- |

Mean score: 4.7

% score≥4: 100%

STRONG CONSENSUS

| M6 | For patients with PDCD who are fasting >24 hours (including surgery/anaesthesia), parenteral nutrition containing glucose (≤5%), lipids, and amino acids should be administered. GRADE BI |
| --- | --- |

| M7 | PDCD is not yet a curable disease, but specific and personalized management can greatly help reduce symptoms and ease the disease burden. The specific approach depends on the patient and the specific disease (phenotype and genotype). Molecular genetic confirmation of the PDCD diagnosis is necessary to implement a personalized treatment plan. GRADE BI |
| --- | --- |

| M8 | Most patients with PDCD need multiple therapies including KD, thiamine and symptomatic therapies e.g. antiseizure medications and other therapies where indicated. GRADE BI |
| --- | --- |

Mean score: 4.7

% score≥4: 100%

STRONG CONSENSUS

Mean score: 4.4

% score≥4: 94%

STRONG CONSENSUS

Mean score: 4.1

% score≥4: 88%

STRONG CONSENSUS

| M9 | For seizure control, good clinical practice including general indications, contraindications, clinical monitoring and side effects for all antiseizure medications must always be kept in mind. GRADE CI |
| --- | --- |

Mean score: 4.7

% score≥4: 100%

STRONG CONSENSUS

| M10 | Topiramate, due to higher risk of precipitating acidosis, should not be considered a first-line treatment for seizures in PDCD, although its use in specific cases is not absolutely contraindicated. GRADE BI |
| --- | --- |

| M11 | Caution should be exercised when considering the use of propofol to treat refractory status epilepticus in PDCD due to the risk of propofol infusion syndrome. GRADE CI |
| --- | --- |

| M12 | In patients with PDCD, medications that can induce acidosis should be avoided. If their use is unavoidable, they must be administered with close clinical and biochemical monitoring. GRADE CIIa |
| --- | --- |

Mean score: 4.6

% score≥4: 100%

STRONG CONSENSUS

Mean score: 4.1

% score≥4: 88%

STRONG CONSENSUS

Mean score: 4.2

% score≥4: 93%

STRONG CONSENSUS

| M13 | In the advanced stages of the disease, or when the disease burden requires it, access to palliative care services is valuable for managing symptoms, improving quality of life, and supporting emotional and family needs. GRADE CI |
| --- | --- |

Mean score: 4.9

% score≥4: 100%

STRONG CONSENSUS

| M14 | It is important to remain vigilant in preventing and promptly managing respiratory infections in PDCD, with families actively involved in monitoring for warning signs. GRADE AI |
| --- | --- |

| M15 | Patients with PDCD should have a swallowing evaluation by a specialized team if there is a clinical concern. GRADE AI |
| --- | --- |

| M16 | Diet modifications and other compensatory techniques can help manage dysphagia, support caloric intake, and potentially reduce aspiration risk. GRADE AI |
| --- | --- |

Mean score: 4.9

% score≥4: 100%

STRONG CONSENSUS

Mean score: 4.4

% score≥4: 89%

STRONG CONSENSUS

Mean score: 4.2

% score≥4: 82%

STRONG CONSENSUS

| M17 | In patients with diagnosed dysphagia or frequent respiratory infections or aspiration pneumonia, the use of a nasogastric tube or surgical or endoscopic placement of a feeding tube should be considered promptly to prevent complications and improve nutritional status and outcomes. GRADE AI |
| --- | --- |

Mean score: 4.6

% score≥4: 94%

STRONG CONSENSUS

| M18 | Palliative and complex care teams should be integrated early in the clinical course, to provide essential guidance, emotional support, and symptom and therapeutic management, helping families navigate the complexities of care. This proactive involvement enhances the patient’s and their family’s quality of life and empowers caregivers to make well-informed decisions regarding treatment and comfort measures. GRADE CI |
| --- | --- |

| M20 | Thiamine supplementation 150-1000 mg/day ideally in three divided doses is recommended in patients with PDCD. GRADE BI |
| --- | --- |

| M19 | An advanced palliative care plan including end-of-life considerations should be established early, clearly outlining goals of care, anticipated complications, and patient and family preferences. GRADE CI |
| --- | --- |

 =

Mean score: 4.3

% score≥4: 89%

STRONG CONSENSUS

Mean score: 4.4

% score≥4: 89%

STRONG CONSENSUS

Mean score: 4.6

% score≥4: 94%

STRONG CONSENSUS

| M21 | For *PDHA1* or *PDHB* variants of unknown thiamine-responsiveness, a dose-dependent response of fibroblast-based PDC activity to thiamine pyrophosphate may be considered to identify thiamine-responsive PDCD. There are a number of known *PDHA1* variants that are thiamine-responsive. GRADE BI |
| --- | --- |

Mean score: 3.8

% score≥4: 75%

CONSENSUS

| M22 | Thiamine supplementation may help control paroxysmal symptoms with proper long-term adherence, reduce the frequency of paroxysmal episodes in PDCD. GRADE BI |
| --- | --- |

| M23 | Apart from thiamine, evidence supporting the use of other vitamins and cofactors in PDCD remains limited. GRADE C |
| --- | --- |

| M24 | L-carnitine deficiency is a possible complication of ketogenic diet. L-carnitine supplementation may be considered if low levels of free carnitine are observed in bloodspot or plasma. GRADE CIIa |
| --- | --- |

Mean score: 4.2

% score≥4: 89%

STRONG CONSENSUS

Mean score: 3.9

% score≥4: 75%

CONSENSUS

Mean score: 4.7

% score≥4: 94%

STRONG CONSENSUS

| M25 | Metabolic acidosis is a possible complication of ketogenic diet.  Bicarbonate, and/or citrate supplementation may be needed to treat metabolic acidosis in patients with PDCD. GRADE CIIa |
| --- | --- |

Mean score: 4.2

% score≥4: 83%

STRONG CONSENSUS

| M27 | DCA has been shown to be safe and well tolerated in patients with PDCD following acute intravenous or chronic oral use. GRADE BIIa |
| --- | --- |

Mean score: 4

% score≥4: 75%

STRONG CONSENSUS

Mean score:4

% score≥4: 82%

STRONG CONSENSUS

| M28 | The recommended DCA dosing depends on the *EGT* haplotype of *GSTZ1*, distinguishing fast metabolizers (25 mg/kg/day) from slow metabolizers (12.5 mg/kg/day). GRADE BIIa |
| --- | --- |

| M26 | Dichloroacetate (DCA) is an investigational drug. It is an activator of the PDC and works by inhibiting pyruvate dehydrogenase kinase. It helps to reduce lactic acid levels in both plasma and CSF. GRADE CIIa |
| --- | --- |

Mean score: 4.3

% score≥4: 87%

STRONG CONSENSUS

| M29 | Clinical benefits of DCA in patients with PDCD remain to be established, pending the results of a recently completed randomized controlled trial. GRADE C |
| --- | --- |

Mean score: 4.6

% score≥4: 100%

STRONG CONSENSUS

| M30 | It is essential to provide patients with PDCD an emergency management letter outlining their condition and suggested management of key complications, including special recommendations for those on KD. GRADE CI  Mean score: 4.7  % score≥4: 94%  STRONG CONSENSUS |
| --- | --- |

| M31 | Acute metabolic decompensation events in PDCD may be precipitated by periods of metabolic stress including fasting, insufficient food intake, inappropriately high carbohydrate intake, vomiting, diarrhoea, intense physical exercise, fever, intercurrent illness, anaesthesia and surgery. GRADE B |
| --- | --- |

| M32 | The most typical biochemical findings of metabolic decompensation events in PDCD include worsening of lactate (above usual baseline), metabolic acidosis and excessive ketosis. GRADE B |
| --- | --- |

Mean score: 4.4

% score≥4: 88%

STRONG CONSENSUS

Mean score: 4.7

% score≥4: 94%

STRONG CONSENSUS

| M33 | Symptoms of metabolic decompensation in PDCD include worsening of neurological status (confusion, lethargy, seizure, focal neurological signs), laboured or rapid breathing, fatigue, difficulty ambulating, fast heart rate, headache and vomiting. GRADE B |
| --- | --- |

Mean score: 4.5

% score≥4: 94%

STRONG CONSENSUS

23

Mean score: 4.4

% score≥4: 88%

STRONG CONSENSUS

Mean score: 4.4

% score≥4: 94%

STRONG CONSENSUS

| M35 | The metabolic tests include: blood gas, glucose, electrolytes, bicarbonate, liver and kidney function, lactate, ketones.  The general blood and urine tests include CBC, CRP, blood cultures if febrile, urinalysis. GRADE CI |
| --- | --- |

| M34 | When acute metabolic decompensation is suspected clinically, peripheral venous catheterisation should be undertaken and blood tests obtained before starting IV fluids. GRADE CI |
| --- | --- |

| M36 | During acute metabolic decompensation events in PDCD, medical therapy should be concurrently instituted to treat any underlying intercurrent illness. GRADE CI |
| --- | --- |

Mean score: 4.8

% score≥4: 100%

STRONG CONSENSUS

| M37 | In cases of elevated lactate above the individual’s baseline, particularly during acute illness, secondary causes such as severe infection, sepsis, or cardiorespiratory instability must be promptly excluded. GRADE AI |
| --- | --- |

Mean score: 4.4

% score≥4: 78%

STRONG CONSENSUS

| M39 | During management of metabolic decompensation, use age-appropriate saline-based IV fluids but if there are concerns regarding hypoglycaemia or prolonged IV use, consider adding low dextrose fluids (e.g. to a final concentration of 2.5% dextrose). GRADE CI |
| --- | --- |

Mean score: 4.7

% score≥4: 100%

STRONG CONSENSUS

| M38 | Where hydration is required, aim to use fluids containing the lowest possible dextrose level, while maintaining blood glucose levels  ≥2.5mmol/L or ≥45mg/dL. Extreme vigilance should be used providing dextrose containing fluids whether or not the patient is on KD. GRADE CI |
| --- | --- |

| M40 | Ringer lactate (also known as [lactated Ringer's solution](https://www.google.com/search?q=lactated+ringer%27s+solution&rlz=1C1GCEA_enGB1141GB1141&oq=Ringer+lactate&gs_lcrp=EgZjaHJvbWUqBwgAEAAYgAQyBwgAEAAYgAQyBwgBEAAYgAQyBwgCEAAYgAQyBwgDEAAYgAQyBwgEEAAYgAQyBwgFEAAYgAQyBwgGEAAYgAQyBwgHEAAYgAQyBwgIEAAYgAQyBwgJEAAYgATSAQgxNDQ1ajBqN6gCALACAA&sourceid=chrome&ie=UTF-8&ved=2ahUKEwi_tvDDtMaQAxWYVUEAHVBoAbYQgK4QegYIAQgAEAU) or Hartmann's solution) is contraindicated in PDCD. GRADE BI |
| --- | --- |

Mean score: 4.5

% score≥4: 100%

STRONG CONSENSUS

Mean score: 4.8

% score≥4: 100%

STRONG CONSENSUS

| M41 | If there is metabolic acidosis (pH <7.20) and/or bicarbonate <10mmol/L and/or base deficit >10mmol/L, then IV bicarbonate (or citrate) could be considered. GRADE BI |
| --- | --- |

Mean score: 4.3

% score≥4: 93%

STRONG CONSENSUS

25

| M42 | If there is ketosis >5mmol/L in acute metabolic decompensation in PDCD, IV glucose should be avoided, and patients should be hydrated using normal saline / electrolyte-balanced crystalloids to decrease ketone levels. GRADE CI |
| --- | --- |

| M43 | During periods of fasting for elective procedures or during acute illness when feeds are not being tolerated, metabolic monitoring of glucose and ketones and blood gas should be undertaken 4 hourly. GRADE CI |
| --- | --- |

| M44 | Patients on KD can typically tolerate low blood sugar levels, symptomatic hypoglycaemia is more likely when glucose levels are <2.5mmol/L. IV glucose should be administered when hypoglycaemia occurs. GRADE AI |
| --- | --- |

Mean score: 4.1

% score≥4: 83%

STRONG CONSENSUS

Mean score: 4.6

% score≥4: 100%

STRONG CONSENSUS

Mean score: 3.8

% score≥4: 75%

CONSENSUS

| M45 | Severe acute decompensations of PDCD require management in an intensive care unit. GRADE BI |
| --- | --- |

Mean score: 4.2

% score≥4: 76%

STRONG CONSENSUS

| M46 | EEG and MRI are valuable tools for diagnosing and managing neurological manifestations in severe acute decompensations of PDCD. GRADE BI |
| --- | --- |

| M47 | Slow titration and dose adjustment of volatile and parenteral anaesthetics are recommended in PDCD to minimize adverse effects. GRADE CI |
| --- | --- |

Mean score: 4.3

% score≥4: 94%

STRONG CONSENSUS

Mean score: 4.5

% score≥4: 93%

STRONG CONSENSUS

| M48 | Prolonged use of propofol during maintenance anaesthesia may elevate the risk of lactic acidosis in PDCD. GRADE BI |
| --- | --- |

Mean score: 4.3

% score≥4: 100%

STRONG CONSENSUS

| M49 | Neuromuscular blocking agents should be avoided in individuals with muscle disease or used only under strict monitoring if absolutely necessary. GRADE BI |
| --- | --- |

| M50 | Stressors, such as changes in body temperature, catabolism, and drugs that exacerbate lactic acidosis (including lactate-containing agents), should be avoided perioperatively in PDCD. GRADE BI |
| --- | --- |

| M51 | Close monitoring of acid-base balance, glucose, ketone bodies, and lactate levels is essential during the perioperative period in patients with PDCD. GRADE BI |
| --- | --- |

Mean score: 4.6

% score≥4: 100%

STRONG CONSENSUS

Mean score: 4.6

% score≥4: 100%

STRONG CONSENSUS

| M52 | Fasting and the use of intravenous glucose during the perioperative period in prolonged anaesthesia should be individualized in patients with PDCD, particularly for those on ketogenic diet. GRADE CI |
| --- | --- |

Mean score: 4.6

% score≥4: 100%

STRONG CONSENSUS

Mean score: 4.1

% score≥4: 93%

STRONG CONSENSUS

| M53 | Based on the available evidence we recommend the use of ketogenic diet (KD) in the management of PDCD. GRADE CI |
| --- | --- |

Mean score: 4.9

% score≥4: 100%

STRONG CONSENSUS

| M54 | We recommend that KD should be used in the management of other genetic forms of primary PDCD (*PDHB*, *DLAT*, *PDHX*, and *PDP1*) given the shared underlying pathophysiology. although the evidence about KD in PDCD largely pertains to patients with *PDHA1* variants. GRADE CI |
| --- | --- |

| M55 | The classical KD is recommended for patients with PDCD but a modified KD could be considered in individual patients e.g. with milder disease, later onset or difficulties with tolerance. GRADE CI |
| --- | --- |

| M56 | KD can be delivered via various modes, including oral, enteral, and parenteral routes. The route should be determined by the patient’s overall clinical condition. GRADE CIIa |
| --- | --- |

Mean score: 4.8

% score≥4: 100%

STRONG CONSENSUS

Mean score: 4.7

% score≥4: 94%

STRONG CONSENSUS

Mean score: 4.6

% score≥4: 100%

STRONG CONSENSUS

| M57 | In PDCD there is no specific target ketone level. Target blood ketone levels are individualised to each patient and are at the level at which they are clinically stable. GRADE C |
| --- | --- |

Mean score: 4.6

% score≥4: 100%

STRONG CONSENSUS

| M58 | KD should be started as soon as feasibly possible following confirmed genetic diagnosis of primary PDCD, regardless of age. GRADE CI |
| --- | --- |

| M59 | KD remains the primary treatment for PDCD and should not be delayed in favour of a thiamine trial, particularly in patients presenting with seizures and severe lactic acidosis. GRADE CI |
| --- | --- |

| M60 | KD often leads to a decrease in lactate levels in PDCD, although the response may vary. GRADE C |
| --- | --- |

Mean score: 4.8

% score≥4: 100%

STRONG CONSENSUS

Mean score: 4.9

% score≥4: 100%

STRONG CONSENSUS

Mean score: 4.4

% score≥4: 94%

STRONG CONSENSUS

| M61 | There is some evidence in the published literature of improvement in quality of sleep after implementation of KD. GRADE C |
| --- | --- |

Mean score: 4.1

% score≥4: 89%

STRONG CONSENSUS

| M62 | There is some evidence in the published literature of progression with social interaction after implementation of KD. GRADE C |
| --- | --- |

| M63 | There is some evidence in the published literature of improvement in motor development after implementation of KD. GRADE C |
| --- | --- |

| M64 | For patients with seizures, there is evidence in the published literature that KD is effective in seizure reduction. GRADE C |
| --- | --- |

Mean score: 4.7

% score≥4: 100%

STRONG CONSENSUS

Mean score: 4.2

% score≥4: 94%

STRONG CONSENSUS

Mean score: 4.2

% score≥4: 94%

STRONG CONSENSUS

| M65 | In patients with PDCD, following initiation of KD, there may be improvement in seizures, ataxia, Leigh syndrome spectrum decompensation episodes, movement disorders, motor development and social interaction. There may be minor improvement in sleep but no normalisation in sleep behaviour. There may a decrease in hospital admissions. GRADE C |
| --- | --- |

Mean score: 4.7

% score≥4: 100%

STRONG CONSENSUS

| M66 | KD may help control paroxysmal symptoms during the acute phase and, with proper long-term adherence, reduce the frequency of paroxysmal episodes in PDCD. GRADE B |
| --- | --- |

Mean score: 4.3

% score≥4: 100%

STRONG CONSENSUS

| S1 | All patients with highly suspected or confirmed PDCD disease should be referred to a centre of expertise. GRADE BI |
| --- | --- |

Mean score: 4.8

% score≥4: 100%

STRONG CONSENSUS

Mean score: 4.8

% score≥4: 100%

STRONG CONSENSUS

Mean score: 4.7

% score≥4: 100%

STRONG CONSENSUS

**SURVEILLANCE**

Mean score:4.8

% score≥4: 100%

STRONG CONSENSUS

| S2 | A rare disease specialist (inherited metabolic disease specialist, geneticist, paediatric/adult neurologist) with clinical experience in PDCD supported by a local multidisciplinary team (including at minimum a dietitian with experience in ketogenic diet), should lead the patient’s care. GRADE CI |
| --- | --- |

| S3 | A care and assessment plan should be individualized to the patient and healthcare context, integrated at the beginning of care and be regularly updated according to the patient’s clinical course and needs. GRADE BI |
| --- | --- |

| S4 | Psychosocial support should be offered (where available) to families after diagnosis. GRADE CI |
| --- | --- |

| S5 | Families should be informed about local and/or international patient organisations that offer support for PDCD. GRADE CI |
| --- | --- |

Mean score: 4.8

% score≥4: 100%

STRONG CONSENSUS

| S6 | Families should be informed of clinical trials for PDCD locally and internationally and provided links to trial information e.g. clinicaltrials.gov in USA, euclinicaltrials.eu in the EU or equivalent in other regions. GRADE CI |
| --- | --- |

| S7 | Implementation of regular physical, speech/swallow, occupational and other supportive therapies should be recommended and tailored to patients’ specific needs to prevent complications and to preserve optimal functioning and quality of life. GRADE A1 |
| --- | --- |

| S8 | Regular laboratory assessments are recommended to enable early recognition of complications caused by the disease or its therapy, as well as to assess nutritional status. GRADE CI |
| --- | --- |

Mean score: 4.4

% score≥4: 89%

STRONG CONSENSUS

Mean score: 4.9

% score≥4: 100%

STRONG CONSENSUS

Mean score: 4.7

% score≥4: 100%

STRONG CONSENSUS

| S9 | Although they should be adjusted to the individual patient’s needs, we recommend the following laboratory tests and intervals.  For neonates and infants depending on their metabolic stability, either every 3 months or at least every 6 months.  For older children (<12 yo), we recommend an interval of every 6-12 months.  For teenagers and adults, we recommend an interval of once per year. GRADE CI |
| --- | --- |

Mean score: 4.2

% score≥4: 83%

STRONG CONSENSUS

| S10 | Core Parameters for laboratory monitoring include the following:  Full blood count with differential, electrolytes, transaminases and liver function tests, kidney function (urea, creatinine, cystatin c and cystatin based eGFR if possible in patients with renal stones, impaired kidney function and/ or low muscle mass), blood lactate, full blood gas, plasma/serum amino acids, ferritin, micronutrients. GRADE CIIa |
| --- | --- |

Mean score: 4

% score≥4: 83%

STRONG CONSENSUS

| S11 | Additional parameters for monitoring (in particular for patients on ketogenic diet) include blood beta-hydroxybutyrate, pancreatic function with lipase (and amylase), lipid panel (triglycerides and cholesterol), plasma free and total carnitine, and a urine dipstick. GRADE CIIa |
| --- | --- |

Mean score: 4

% score≥4: 72%

STRONG CONSENSUS

| S12 | Nutritional status of patients with PDCD should be closely monitored. Anthropometrics (weight, height, head circumference, growth velocity, in infants and children) should be assessed at each follow up. GRADE CI |
| --- | --- |

Mean score: 4.8

% score≥4: 100%

STRONG CONSENSUS

| S13 | Assessment of nutritional status should not only rely on weight and height measurements but should include regular monitoring of other physical signs of undernutrition (e.g. muscle wasting, loss of body fat) and measurement of micronutrients (e.g. protein/ albumin, plasma/serum amino acids, iron status, 25-hydroxy vitamin D, parathyroid hormone, vitamin B12, folate, calcium, phosphate, magnesium, selenium, zinc) at least annually. GRADE BIIa |
| --- | --- |

| S14 | Careful attention should be paid to ensure adequate hydration particularly those on KD as PDCD patients may have a reduced ability to communicate thirst and may experience decreased fluid intake due to swallowing difficulties and losses due to drooling. GRADE BI |
| --- | --- |

| S15 | Hydration needs should be adjusted for patients on a ketogenic diet. GRADE B1 |
| --- | --- |

Mean score: 4.2

% score≥4: 83%

STRONG CONSENSUS

Mean score: 4.6

% score≥4: 94%

STRONG CONSENSUS

Mean score: 4.3

% score≥4: 83%

STRONG CONSENSUS

| S16 | A percutaneous enteral gastrostomy (PEG) feeding tube is recommended for individuals unable to meet nutritional requirements, those at high risk of aspiration or with confirmed silent aspiration on video fluoroscopy, or those experiencing recurrent aspiration pneumonia. Discussion should be started early regarding insertion of PEG tube as this is the optimal mode of delivery of enteral feeding. GRADE BI |
| --- | --- |

Mean score: 4.4

% score≥4: 83%

STRONG CONSENSUS

| S17 | Gastrointestinal dysmotility is a common issue in PDCD and may present as delayed gastric emptying (gastroparesis) manifesting with symptoms such as nausea, reflux, vomiting, abdominal bloating or discomfort after meals. Clinical suspicion should prompt referral to a gastroenterologist for further investigation (e.g. gastric emptying studies). GRADE CI |
| --- | --- |

| S18 | Constipation is frequent, often secondary to reduced mobility and underlying gastrointestinal dysmotility. It can also be exacerbated by dietary changes (e.g., ketogenic diet), medications, or low fluid intake. Constipation should be assessed routinely and managed early with standard of care (e.g. fibre supplement, osmotic agents). GRADE CI |
| --- | --- |

Mean score: 4.7

% score≥4: 100%

STRONG CONSENSUS

Mean score: 4.4

% score≥4: 94%

STRONG CONSENSUS

Mean score: 4.4

% score≥4: 94%

STRONG CONSENSUS

Mean score: 4.4

% score≥4: 88%

STRONG CONSENSUS

| S19 | Gastroesophageal reflux disease (GERD) is very common in PDCD patients with neurological impairments and can contribute to feeding difficulties, vomiting, or respiratory symptoms due to aspiration. GERD should be suspected in individuals with frequent regurgitation, feeding refusal, chronic cough, or unexplained irritability. Management may include pharmacologic therapy, dietary modifications and surgical intervention. GRADE BI |
| --- | --- |

| S20 | A trial of antireflux therapy (e.g. proton pump inhibitors) should be considered in patients with suspected GERD or unexplained signs of pain. GRADE BI |
| --- | --- |

| S21 | Neurodevelopmental and cognitive status should be closely monitored at each follow-up since individuals with PDCD are at risk of developmental disabilities and neurological regression. GRADE BI |
| --- | --- |

Mean score: 4.8

% score≥4: 100%

STRONG CONSENSUS

| S22 | A standardized neuropsychological or psychoeducational assessment appropriate for age is indicated at the time of diagnosis for individuals with PDCD. Repeated testing is recommended based on suspected changes in functioning, disease progression, and for monitoring efficacy of treatment. GRADE CI |
| --- | --- |

Mean score: 4.4

% score≥4: 88%

STRONG CONSENSUS

| S24 | Individuals with PDCD and speech impairment should be evaluated by a speech and language therapist for the possible need of speech and augmentative communication therapy. GRADE CI |
| --- | --- |

| S23 | Developmental regression at any age warrants a thorough evaluation for a potentially treatable disease complication as well as an assessment for disease progression and evaluation of the ongoing treatment regimen/ ketogenic diet. Testing may need to include repeat neuroimaging and an electroencephalogram where there are concerns that there may be subclinical seizures. GRADE BIIa |
| --- | --- |

Mean score: 4.8

% score≥4: 94%

STRONG CONSENSUS

Mean score: 4.7

% score≥4: 100%

STRONG CONSENSUS

| S25 | Individuals with PDCD and global developmental delay/ intellectual disability (ID) require special educational services and individualized education plans in school. Referrals to a developmental specialist and appropriate therapist, and for community and school support services should be considered. Anticipatory guidance should be provided to parents, siblings, and the family. GRADE BI |
| --- | --- |

Mean score: 4.7

% score≥4: 100%

STRONG CONSENSUS

| S26 | Individuals with PDCD are at increased risk for neuropsychiatric and mental health issues. Depending on the age, the patient should be screened for autism and attention deficit disorder with or without hyperactivity, and/or psychiatric symptoms, e.g. depression and anxiety. Referral for psychiatric evaluation is recommended in positively screened patients. Individual and family support as well as behavioural and pharmacological therapy should be initiated according to standard practices. GRADE BI |
| --- | --- |

| S27 | Pharmacological treatment of seizures in PDCD should follow general guidelines for epilepsy treatment similar to other mitochondrial disorders, according to the guidelines published by the InterERN Mitochondrial Working Group. GRADE BI |
| --- | --- |

Mean score: 4.9

% score≥4: 100%

STRONG CONSENSUS

Mean score: 4/6

% score≥4: 100%

STRONG CONSENSUS

| S28 | Good clinical practice must always be kept in mind for all antiseizure medications, including general indications, contraindications, clinical monitoring and side effects. GRADE CI |
| --- | --- |

Mean score: 4.9

% score≥4: 100%

STRONG CONSENSUS

| S29 | Individuals with PDCD who develop seizures should be referred to a specialist in neurology for advice about epilepsy management. GRADE CI |
| --- | --- |

| S30 | Seizures in PDCD are predominantly generalized but may be focal in onset and are treated with antiseizure medications based on standard practice according to seizure semiology and EEG findings. GRADE BI |
| --- | --- |

| S31 | KD is an efficient treatment option for the underlying disease and has been shown to be an effective treatment for epileptic seizures in PDCD. GRADE BI |
| --- | --- |

Mean score: 4.7

% score≥4: 100%

STRONG CONSENSUS

Mean score: 4.8

% score≥4: 100%

STRONG CONSENSUS

Mean score: 4.9

% score≥4: 100%

STRONG CONSENSUS

| S32 | Epileptic spasms are frequently seen in infants with PDCD. We recommend an EEG awake and asleep or early detection of hypsarrhythmia or other EEG abnormalities, with referral to a paediatric neurologist if indicated. GRADE BI |
| --- | --- |

Mean score: 4

% score≥4: 100%

STRONG CONSENSUS

| S33 | If infantile spasms or hypsarrhythmia is detected in an infant PDCD then the first line management is KD rather than steroids. GRADE CI |
| --- | --- |

| S34 | No ASM is absolutely contraindicated in PDCD. Caution should be taken for ASMs that are known to be associated with metabolic acidosis (e.g. topiramate, zonisamide). GRADE CI |
| --- | --- |

| S35 | An acute management plan for seizure deterioration and status epilepticus is needed. GRADE CI |
| --- | --- |

Mean score: 4.4

% score≥4: 89%

STRONG CONSENSUS

Mean score: 4

% score≥4: 100%

STRONG CONSENSUS

Mean score: 4

% score≥4: 100%

STRONG CONSENSUS

| S36 | The management plan for worsening seizures should include review of the KD formulation, together with a specialist dietitian. GRADE CI |
| --- | --- |

Mean score: 4.5

% score≥4: 100%

STRONG CONSENSUS

| S37 | Any antiseizure medications should be of a carbohydrate-free formulation, particularly in individuals with PDCD on KD. GRADE BI |
| --- | --- |

| S38 | Neurological assessment for disorders of movement and tone is needed at each outpatient visit. GRADE BI |
| --- | --- |

| S39 | Sudden onset of a new disorder of movement or tone should lead to an evaluation of disease progression, treatment failure, and potential secondary causes. Baseline laboratory investigations and neuroimaging are recommended. GRADE CI |
| --- | --- |

Mean score: 4.7

% score≥4: 94%

STRONG CONSENSUS

Mean score: 4.8

% score≥4: 100%

STRONG CONSENSUS

Mean score: 4.5

% score≥4: 94%

STRONG CONSENSUS

| S40 | Management of movement and tone disorders in PDCD is similar to patients with movement disorders of other causes. Medical, procedural, and surgical treatments and review of KD should be considered. Treatment can improve quality of life. GRADE CI |
| --- | --- |

Mean score: 4.9

% score≥4: 83%

STRONG CONSENSUS

| S41 | Caution should be used with medications that alter tone (such as baclofen, benzodiazepines, anticholinergic medication such as trihexyphenidyl) as they can selectively worsen level of consciousness, decrease muscle strength and respiratory effort, and impact GI motility and bladder function. GRADE CI |
| --- | --- |

| S42 | Individuals with PDCD should receive physical and occupational therapy input commensurate to the severity of their motor function deficits, to maximize gross and fine motor function including mobility, prevent contractures, joint subluxations/dislocations, and alleviate discomfort and pain. GRADE BI |
| --- | --- |

| S43 | With genotype-based dosing of DCA, there is no need to undertake additional nerve conduction study (NCS) in PDCD patients commenced on DCA, unless peripheral neuropathy is suspected clinically. GRADE CI |
| --- | --- |

Mean score: 5

% score≥4: 100%

STRONG CONSENSUS

Mean score: 4.8

% score≥4: 94%

STRONG CONSENSUS

Mean score: 4.5

% score≥4: 93%

STRONG CONSENSUS

| S44 | An NCS (together with an electromyogram) may be considered if clinical symptoms/signs are suggestive of peripheral nerve dysfunction or in case of unexplained complaints (e.g. pain, if more common causes have been excluded). The nerve conduction study should include both upper and lower extremities with evaluation of both sensory and motor nerves. GRADE CIIb |
| --- | --- |

Mean score: 4.5

% score≥4: 93%

STRONG CONSENSUS

| S45 | Treatment of peripheral neuropathy in PDCD is symptomatic and follows standard of care established for other neuropathies. GRADE CI |
| --- | --- |

| S46 | Neuroimaging with MRI including diffusion-weighted imaging (and ideally with spectroscopy) should be performed at the time of diagnosis. Repeated imaging should be considered if new neurological symptoms occur or progression/ deterioration of symptoms is noted. GRADE CIIa |
| --- | --- |

| S47 | In patients with PDCD, a baseline echocardiogram and electrocardiogram should be considered and repeat cardiac assessment should be guided by a cardiologist.  Patients with postural abnormalities should also receive echocardiographic monitoring. Cardiac examinations should be guided by the specific needs or symptoms of the patient, particularly in relation to the potential cardiovascular effects of medications received. GRADE CIIb |
| --- | --- |

Mean score: 4.1

% score≥4: 94%

STRONG CONSENSUS

Mean score: 4.4

% score≥4: 94%

STRONG CONSENSUS

Mean score: 4.7

% score≥4: 100%

STRONG CONSENSUS

| S48 | Respiratory compromise in PDCD may result from a combination of hypotonia, impaired central respiratory control, bulbar dysfunction, reduced mobility and posture abnormalities like scoliosis, leading to ineffective airway clearance, hypoventilation, sleep-disordered breathing, and increased risk of aspiration pneumonia. GRADE BI |
| --- | --- |

Mean score: 4.7

% score≥4: 100%

STRONG CONSENSUS

45

| S49 | Baseline and ongoing respiratory evaluation should be part of routine care, especially in patients with hypotonia, scoliosis, feeding difficulties, and/or recurrent respiratory infections. GRADE BI |
| --- | --- |

| S50 | Assessment may include clinical exam, pulse oximetry, capnography, or overnight oximetry/polysomnography if there are concerns about sleep-disordered breathing or nocturnal hypoventilation. Referral to a respiratory/ pulmonary specialist is recommended if abnormalities are detected or recurrent respiratory infections occur. GRADE CI |
| --- | --- |

Mean score: 4.7

% score≥4: 100%

STRONG CONSENSUS

| S51 | Preventive strategies include vaccinations (e.g. influenza, pneumococcal), appropriate management of feeding to reduce risk of aspiration, chest physiotherapy and secretion management including mechanical cough-assist devices as needed. GRADE CI |
| --- | --- |

Mean score: 4.8

% score≥4: 100%

STRONG CONSENSUS

Mean score: 4.7

% score≥4: 100%

STRONG CONSENSUS

| S52 | Non-invasive ventilation may be indicated for patients with sleep-disordered breathing or (nocturnal) hypoventilation. GRADE CI |
| --- | --- |

Mean score: 4.6

% score≥4: 100%

STRONG CONSENSUS

Mean score: 4.3

% score≥4: 80%

STRONG CONSENSUS

| S53 | Invasive ventilation (via tracheostomy) may be necessary in patients with recurrent aspiration pneumonia, respiratory insufficiency and/ or recurrent central apnoea.  Risks and benefits should be discussed early and thoroughly with families before such invasive ventilation intervention is commenced. GRADE CIIb |
| --- | --- |

| S54 | Decisions should be integrated into a comprehensive palliative care plan, including consideration of ceilings of care and Do Not Resuscitate orders. GRADE CI |
| --- | --- |

| S55 | At diagnosis and in routine follow up, clinical examination should include assessment of spinal abnormalities including scoliosis, kyphosis/lordosis, joint subluxation (in particular the hips due to hip dysplasia), talipes equinovarus deformity of the feet. GRADE BI |
| --- | --- |

Mean score: 4.7

% score≥4: 100%

STRONG CONSENSUS

Mean score: 4.7

% score≥4: 100%

STRONG CONSENSUS

| S56 | Symptoms including pain, changes to mobility, and posture should be ascertained in order to assess the impact of these clinical examination findings. GRADE CI |
| --- | --- |

Mean score: 4.7

% score≥4: 100%

STRONG CONSENSUS

| S57 | Referral to orthopaedics for consideration of surgical options should be made following identification of bone and joint abnormalities throughout life, utilising plain film radiographs as a first line; additional imaging may be indicated following this, as advised by the orthopaedic team. GRADE BI |
| --- | --- |

| S58 | Referral to physiotherapist, occupational therapist, podiatrist, and wheelchair service where appropriate should also be initiated. GRADE BI |
| --- | --- |

| S59 | Non-surgical options including (but not limited to) ankle orthoses, spinal braces, mobility aids and surgical options including (but not limited to) femoral or acetabular osteotomies, Achilles and other tendon release surgery, spinal surgery for scoliosis, should be discussed with the family by the orthopaedic team, if relevant for the individual patient’s clinical problems.  GRADE CI |
| --- | --- |

Mean score: 4.8

% score≥4: 100%

STRONG CONSENSUS

Mean score: 4.7

% score≥4: 100%

STRONG CONSENSUS

Mean score: 4.4

% score≥4: 88%

STRONG CONSENSUS

| S60 | Surgical decision making should consider multidisciplinary opinions to weigh potential benefits of surgery against general, specific and anaesthetic risks. All patients having surgery should have a perioperative plan which should be agreed by the lead clinical team (inherited metabolic disease/ neurology/ genetics as appropriate). GRADE CI |
| --- | --- |

Mean score: 4.8

% score≥4: 100%

STRONG CONSENSUS

| S61 | Owing to the risk of osteoporosis due to long term immobilisation in many patients, biochemical assessment of bone health including bone profile, parathyroid hormone and 25-hydroxy vitamin D should be undertaken. Bone densitometry studies (DEXA) should be considered if there is a history of fragility (atraumatic) fractures. Referral to a specialist in bone metabolism should be initiated following the identification of a fragility fracture for consideration of bone protection therapy. GRADE CI |
| --- | --- |

| S62 | Ketogenic diet increases the risk of kidney stone formation, with potential complications such as obstructive uropathy, acute and/or chronic kidney disease. The estimated incidence of kidney stones in patients on ketogenic diets is approximately 5.9% but may be even higher in patients with PDCD due to underlying metabolic acidosis. Reported stone types include uric acid stones (49%), calcium oxalate or calcium phosphate stones (36%), or mixed uric-acid and calcium-based stones. GRADE B |
| --- | --- |

Mean score: 4.6

% score≥4: 100%

STRONG CONSENSUS

Mean score: 4.6

% score≥4: 100%

STRONG CONSENSUS

| S63 | Preventive and monitoring strategies to reduce the risk of kidney stone formation in patients with PDCD, especially if on ketogenic diet include:   - Adequate hydration – to maintain dilute urine and reduce crystallization risk - Potassium citrate supplementation can alkalinize the urine and decrease calcium stone formation by binding urinary calcium and reducing urinary citrate depletion. - Regular urine analysis: detection of haematuria, crystalluria, monitor hydration - Ultrasound imaging: may be indicated periodically in high-risk patients or in those with unexplained pain or urinary symptoms as guided by a nephrologist/ urologist. GRADE CIIa |
| --- | --- |

Mean score: 4.4

% score≥4: 89%

STRONG CONSENSUS

| S64 | Renal tubular acidosis (RTA) is a recognized manifestation of primary mitochondrial disease and has been reported in individual cases with PDCD. While rare, RTA should be considered in cases of metabolic acidosis that appear disproportionate to lactate levels and are not explained by other common causes such as ketosis. GRADE CI |
| --- | --- |

| S65 | Excessive drooling / sialorrhoea is common in many patients with PDCD and can significantly impact quality of life. If present, families should be advised about avoiding saliva-stimulating substances / drinks. GRADE BI |
| --- | --- |

Mean score: 4.3

% score≥4: 94%

STRONG CONSENSUS

Mean score: 4

% score≥4: 72%

STRONG CONSENSUS

| S66 | There is no contraindication for regular antisialorrhoeic therapy. Thus, as with other patients experiencing sialorrhoea, anticholinergics should be considered as first-line treatment, provided that they are a sugar-free formulation. GRADE BI |
| --- | --- |

Mean score: 4.3

% score≥4: 88%

STRONG CONSENSUS

| S67 | Patients with PDCD often present with abnormal dental status, which may be associated with microcephaly, dysmorphic features, orofacial hypotonia and/or feeding difficulties. Common issues include malocclusion, enamel hypoplasia, delayed eruption/ impaction and increased risk of dental caries. Early and proactive specialist dental care is essential, as well as support for oral hygiene at home and preventive interventions (e.g. fluoride treatments). GRADE CI |
| --- | --- |

| S68 | Sensorineural hearing loss is well described in PDCD. Hyperacusis has also been reported. Concerns regarding a deterioration in hearing should be elicited at routine follow up appointments. All patients with speech delay should be referred for a baseline hearing test. GRADE BI |
| --- | --- |

| S69 | At diagnosis, all patients should have an up-to-date formal age and developmentally appropriate hearing assessment. This may include (but is not limited to): pure tone audiometry, play or visual reinforcement audiometry, tympanometry, auditory brainstem responses and/or otoacoustic emissions.  GRADE CI |
| --- | --- |

Mean score: 4.5

% score≥4: 94%

STRONG CONSENSUS

Mean score: 4.7

% score≥4: 100%

STRONG CONSENSUS

Mean score: 4.64

% score≥4: 94%

STRONG CONSENSUS

| S70 | Consideration of interventions such as hearing aids, cochlear implants, ear defenders and regularity of follow up should be guided by the audiology team. GRADE CI |
| --- | --- |

Mean score: 4.7

% score≥4: 100%

STRONG CONSENSUS

| S71 | All patients with delayed visual or fine motor skills should be referred to an ophthalmologist with expertise in assessing patients with inherited metabolic diseases for formal eye examination. All patients should be referred at diagnosis of PDCD if they have not already been offered ophthalmological assessment. The type of ophthalmological assessment used and follow up interval is to be determined by the ophthalmologist. GRADE CI |
| --- | --- |

| S72 | Surgical and non-surgical management options are to be guided by the ophthalmology team. For those who are registered blind, adjustments should be made to their living environment, care and educational needs. GRADE CI |
| --- | --- |

Mean score: 4.6

% score≥4: 100%

STRONG CONSENSUS

| S73 | Sleep disturbance may substantially worsen daytime function, behaviour and caregiver burden; therefore, assessment of sleep history should be part of routine surveillance. GRADE CI |
| --- | --- |

Mean score: 4.8

% score≥4: 100%

STRONG CONSENSUS

Mean score: 4.6

% score≥4: 100%

STRONG CONSENSUS

| S74 | Where indicated, overnight oximetry and/or polysomnography should be performed, with referral to sleep or respiratory specialists for guidance on airway management, ventilatory support, and therapies. (see respiratory section). GRADE CI |
| --- | --- |

Mean score: 4.8

% score≥4: 100%

STRONG CONSENSUS

| S75 | Reversible or partly treatable contributory factors, including medication effects and timing, pain, mood or behavioural disorders, nocturnal seizures, and dysphagia/ aspiration should be evaluated and managed. GRADE CI |
| --- | --- |

Mean score: 4.7

% score≥4: 100%

STRONG CONSENSUS

| S76 | Non-pharmacological strategies (e.g. sleep hygiene, structured sleep–wake schedules, promotion of daytime activity/physiotherapy, weighted blankets) and medications (e.g., melatonin, sedatives with caution) may help manage sleep disturbances. Medication use should be individualized, carefully weighing potential benefits against risks such as increased hypotonia and nocturnal apnoea. GRADE CIIa |
| --- | --- |

Mean score: 4.7

% score≥4: 94%

STRONG CONSENSUS

S77

Pain should be actively assessed. GRADE CI

Mean score: 4.7

% score≥4: 94%

STRONG CONSENSUS

|  |  |
| --- | --- |
| 5: Absolutely agree | 12 |
| 4: Agree | 6 |
| 3: Neutral | 0 |
| 2: Disagree | 0 |
| 1: Absolutely disagree | 0 |

| S78 | Children with PDCD often have limited language abilities, making pain assessment challenging. Pain may arise from various sources, including osteoporosis-related fractures due to immobility, joint sub-/dislocations, musculoskeletal problems e.g. scoliosis, hip subluxation, increased muscle tone (including dystonia and spasticity), dental issues, urinary retention, pressure ulcers, gastrointestinal problems such as constipation and gastroesophageal reflux including eosinophilic esophagitis. Therefore, patients should be carefully evaluated to identify potential causes of pain and receive appropriate treatment. GRADE CI |
| --- | --- |

Mean score: 4.7

% score≥4: 94%

STRONG CONSENSUS

|  |  |
| --- | --- |
| 5: Absolutely agree | 12 |
| 4: Agree | 6 |
| 3: Neutral | 0 |
| 2: Disagree | 0 |
| 1: Absolutely disagree | 0 |

| T1 | Patients reaching adulthood should ideally be followed by an adult care team, preferably by a specialist experienced in PDCD or related neurometabolic diseases (e.g. adult metabolic medicine/biochemical genetics specialist, adult neurologist, depending on local service provisions) and a dietitian familiar with managing ketogenic diet. GRADE CI |
| --- | --- |

Mean score: 4.8

% score≥4: 100%

STRONG CONSENSUS

**TRANSITION**

| T2 | A holistic approach should be taken in the management of adult patients with PDCD incorporating care by a multidisciplinary team similar to the paediatric setting. GRADE CI |
| --- | --- |

Mean score: 4.9

% score≥4: 100%

STRONG CONSENSUS

| T3 | If a patient is receiving end of life care, continuing care within paediatric services should be considered to minimize additional stress for patients and families. GRADE CI |
| --- | --- |

Mean score: 4.52

% score≥4: 95%

STRONG CONSENSUS

| T4 | Discussion about and planning transition should start at least two years prior to the anticipated transfer to adult care services. GRADE CI |
| --- | --- |

Mean score: 4.6

% score≥4: 95%

STRONG CONSENSUS

54

| T5 | A transition plan with goals and emergency instructions should be developed early during the transition process and should be reviewed at least annually. GRADE CI |
| --- | --- |

Mean score: 4.6

% score≥4: 100%

STRONG CONSENSUS

| T6 | Even though ongoing and lifelong parental or guardian support is often necessary for patients with PDCD, patients should be involved in medical decision-making early and progressively, tailored to their individual abilities. GRADE CI |
| --- | --- |

| T7 | Transition readiness of patient and caregivers should be evaluated and documented in the transition plan, including addressing psychosocial maturity and autonomy of the patient. GRADE CI |
| --- | --- |

Mean score: 4.7

% score≥4: 100%

STRONG CONSENSUS

Mean score: 4.7

% score≥4: 100%

STRONG CONSENSUS

| T8 | Patients should be provided with a skills-based education and tools to increase their autonomy depending on their individual abilities (e.g. pill reminder apps, tablet dispensers, shopping advice for ketogenic diet). GRADE CI |
| --- | --- |

Mean score: 4.6

% score≥4: 100%

STRONG CONSENSUS

| T9 | Where feasible, patients should have the possibility of a transitional period with at least one shared follow up appointment involving both paediatric and adult care. GRADE CIIa |
| --- | --- |

Mean score: 4.6

% score≥4: 95%

STRONG CONSENSUS

| T10 | Patients should not be discharged from paediatric services until they are seen by adult services. GRADE CI |
| --- | --- |

| T11 | The clinician/ service responsible for directing care during emergency department visits for metabolic emergencies must be clearly designated. GRADE CI |
| --- | --- |

| T12 | In the early period following transition, consultation between paediatric and adult physicians is advised for acutely unwell patients. Responsibility for leading acute management is with the adult team; this should be clearly defined and communicated. GRADE CI |
| --- | --- |

Mean score: 4.7

% score≥4: 100%

STRONG CONSENSUS

Mean score: 4.6

% score≥4: 89%

STRONG CONSENSUS

Mean score: 4.7

% score≥4: 100%

STRONG CONSENSUS
